# Use of somatic cell count as an indicator of colostrum quality

**DOI:** 10.1371/journal.pone.0237615

**Published:** 2020-08-11

**Authors:** Kamila Puppel, Marcin Gołębiewski, Grzegorz Grodkowski, Paweł Solarczyk, Piotr Kostusiak, Maria Klopčič, Tomasz Sakowski

**Affiliations:** 1 Institute of Animal Science, Warsaw University of Life Sciences, Warsaw, Poland; 2 Department of Animal Science, Institute of Genetics and Animal Breeding, Polish Academy of Science, Magdalenka, Poland; 3 Department of Animal Science, University of Ljubljana, Domžale, Slovenia; Michigan State University, UNITED STATES

## Abstract

The impact of cow mammary gland diseases on the quality of colostrum is not conclusively defined; research results are conflicting. However, it is widely believed that *mastitis* lowers the level of immunoglobulins and the quality of the colostrum produced. Therefore, the aim of this study was to determine the influence of somatic cell counts (SCC) on the colostrum immunostimulating and chemical components. The experiment was conducted on an experimental organic dairy farm in which a herd of approximately 250 cows was kept in a freestall housing system, with the average performance exceeding 6,000 kg of milk per lactation. Colostrum and milk samples were taken individually from each cow seven times during the experiment: from the first to second day after calving–twice per day, and from the third to fifth day after calving–once per day. Therefore, after preliminary analyses, the cows were divided into two groups based on the cytological quality of their colostrum at the first collection: 1. SCC ≤400,000 cells/ml (good quality colostrum; GCC– 18 cows), 2. SCC *≥* 400,000 cells/ml (low quality colostrum; LCC– 22 cows). The study found almost double the concentration of immunoglobulins and essential fatty acids in first milking colostrum in the GCC group than in colostrum from the LCC group. In addition, an increase in the concentration of lysozyme in first milking colostrum was associated with a decrease in the concentration of immunoglobulins. In addition, the increase in the level of lysozyme was associated with a decrease in the concentration of immunoglobulins. In conclusion, the SCC of first milking colostrum can be used as an indicator of colostrum quality.

## Introduction

The most critical time for calf health is the first two weeks of life, with high mortality rates associated with the feeding of poor quality colostrum, poor environmental hygiene, and digestive disorders [1]. Calves are born essentially agammaglobulinemic and rely on passive absorption of immunoglobulin from colostrum to protect them from disease in the first few weeks of life [2]. During peak transport, more than 550 g IgG per week is actively transported into secretion via epithelial cells and leukocytes [3]. It should be stressed that a calf should receive the first feeding of colostrum up to six hours after birth, and this colostrum should contain approximately 100–200 g of immunoglobulins [4]; any delay in the administration of the first feeding significantly increases the risk of disease and mortality [5,6]. However, adequate passive immunity is generally only achieved when calves are fed high quality colostrum, and production of enough high quality colostrum is a challenge for many modern dairy herds [1].

Colostrum is a rich source of immunity-enhancing components, including immunoglobulins (Ig), lactoferrin (LF), lysozyme (LZ), and polyunsaturated fatty acids (PUFA) [1]. Omega-3 and omega-6 fatty acids exert various biological effects, and some of their activities and functions are related to their transformation products such as eicosanoids. Polyunsaturated fatty acid evoke antibacterial, antiviral, antifungal, and antiparasitic effects [7]. It was discovered that, C18:2 n-6 (LA) and C18:3 n-3 (ALA) deactivate methicillin-resistant *S*. *aureus* strains [8]. ALA supports the adhesion of *Lactobacillus casei* on the surface of the mucosa and stimulates their growth, whereas it reduces the development of pathogenic bacteria from the genera *Helicobacter*, *Shigella*, *Salmonella*, and *Pseudomonas* [9,10]. Lactoferrin is an iron-binding protein with multiple physiological functions: anti-microbial, anti-inflammatory, and immunomodulatory [11]. In contact with Gram (−) bacteria, LF combines with its surface proteins, causing the release of lipopolysaccharide, which results in an increase in membrane permeability, intracellular concentration of antibacterial factors, and intracellular osmotic pressure. [12]. Gram (+) bacteria break down by combining the positively charged proteins with the bacterial membrane. Most often, it is at this stage that the bacterial cell is destroyed [13]. These activities can be as attributable to the direct action of lactoferrin as to a change in bacteria metabolism [11]. Bovine lactoferrin incubated with pork pepsin has eight times higher antibacterial activity than the undigested lactoferrin dose. In this way, a peptide called lactoferricin was formed, which in contact with *E*. *coli* membrane inhibited the attachment of proline to its membrane. The properties of lactoferrin can be further enhanced by their ability to act synergistically with lysozyme [14]. This combination has a destructive effect on *Vibrio cholerae* and *E*. *coli* by swelling and dissolving their cell structure. In addition, LF allows reducing doses of administered antibiotics [15]. Owing to this, in the fight against *Staphylococcus epidermidis*, the administered dose of vancomycin–an antibiotic used to fight this strain–could be reduced twice by lysozyme addition. Likewise, when added to penicillin, it increased its activity even four-fold against *S*. *aureus* [16]. Another useful feature of lactoferrin is its antifungal effect. The fungus cells (*Candidia albicans* and *C*. *krusei*), which were treated with lactoferrin free of iron, changed the structure of the surface by creating blisters on it with leakage of proteins [17].

Recent data show that almost 60 percent of colostrum samples from dairy cows do not have the appropriate level of antibodies to ensure sufficient protection of calves [1,18]. Although a large number of factors have been suggested to influence colostrum quality [19], factors associated with production of poor quality colostrum are not well understood. Ferdowsi et al. [20] suggested that mastitis, as indicated by the somatic cell count (SCC) of colostrum may be one factor that is associated with the quality of colostrum. Maunsell et al. [21] reported that the colostrum from cows with persistent or transient mammary infections differed from the colostrum from uninfected cows. Damaged epithelial cells caused by intramammary infection reduce IgG1 transport and result in low colostral IgG1 concentration in infected glands [22]. Additionally, the reductions of IgG1 contribute markedly to the high incidence of failure of adequate passive transfer of colostral Ig in dairy calves [21]. However, limited information exists on the relationship between SCC and the concentrations of immune-stimulating components in colostrum. The SCC of colostrum is higher than that of milk, and gradually declines over early lactation in uninfected glands [23]. In addition, there are no guidelines for SCC for colostrum. Thus the aim of this study was to determine the association between the SCC and immunostimulating and chemical components of first milking colostrum.

## Material and methods

All cows were handled in accordance with the regulations of the Polish Council on Animal Care, and the Second Ethics Committee for Animal Experimentation in Warsaw reviewed and approved all procedures (Approval number: WAWA2/086/2018). During the experiment, the cows were under veterinary care and remained healthy for the duration of the study and did not show any disorders and diseases that could generate an immune response (e.g., ketosis, acidosis).

### Animals, treatment, and sampling

The experiment was conducted on an experimental organic dairy farm in which a herd of approximately 250 cows was kept in a freestall housing system, with the average performance exceeding 6,000 kg of milk per lactation. Dry cows were fed according to the Nutrient Requirements Committee. Dry cows’ requirements, daily ration, as well as nutrient balances are presented in Tables 1–3.

**Table 1:** Daily requirements of the cows.

Specification	Dry cow groups	
	I	II
**Assumptions**		
Cow weight (kg)	650	680
Pregnancy (days)	220	270
**Maintenance requirements**		
NEL (Mcal/day)	9.9	11.4
Metabolic protein (g/day)	461	656
Ca (g/day)	11	16.5
P (g/day)	12	16.3
K (g/day)	54	55
**Fetus requirements**		
NEL (Mcal/day)	2.9	3
Metabolic protein (g/day)	239	245
Ca (g/day)	4	5
P (g/day)	3	4
K (g/day)	1	2
**Total requirements**		
NEL (Mcal/day)	12.8	14.4
Metabolic protein (g/day)	700	901
Ca (g/day)	15	21.5
P (g/day)	15	20.3
K (g/day)	55	57

**Table 2:** Daily rations of the cows.

Feed (kg/cow/day)	Dry cow groups			
	I (first 5 weeks)		II (last 3 weeks)	
	dry matter (kg)	feed (kg)	dry matter (kg)	feed (kg)
**Roughage:**	**10.99**	**23.50**	**8.79**	**23.10**
Maize silage			4.88	15.00
Alfalfa silage			2.41	6.00
Fgras silage	7.84	20.00		
Corn silage			0.34	0.80
Straw	3.15	3.50	1.17	1.30
**Concentrates:**	**0.65**	**0.65**	**2.48**	**2.80**
Fodder chalk			0.05	0.05
Prophos Trans*	0.15	0.15	0.15	0.15
Rape meal	0.45	0.50	0.53	0.60
Soya meal			0.88	1.00
Grain meal			0.87	1.00

*Ca% 10; F% 8; Na% 6; Mg% 9; Vit A j.m./kg 1 000 000; VIit. D3 j.m./kg 120 000; Vit. E mg/kg 5 000; Vit. B1 mg/kg150; Vit. B2 mg/kg 10; Vit. B6 mg/kg 50; Vit. B12 mg/kg 0,55; Biotin mg/kg 2 500; Folic acid mg/kg 30; Ca, mg/kg 300; Zn mg/kg 9 500; Zn (chelat) mg/kg 4 000; Mn mg/kg 1 150; Se mg/kg 90; Co mg/kg 25; Choline mg/kg 50 000.

**Table 3:** Nutrient balance of the cows.

	Dry cow groups	
	I	II
**Total requirements**		
NEL (Mcal/day)	12.8	14.4
Metabolic protein (g/day)	700	901
Ca (g/day)	15	21.5
P (g/day)	15	20.3
K (g/day)	55	57
**Supply of the nutrients in daily ration**		
NEL (Mcal/day)	13.2	15.1
Metabolic protein (g/day)	750	953
Ca (g/day)	26	58
P (g/day)	22	50
K (g/day)	65	223
**Balance**		
NEL (Mcal/day)	3.03%	4.64%
Metabolic protein (g/day)	6.67%	5.46%
Ca (g/day)	42.31%	62.93%
P (g/day)	31.82%	59.40%
K (g/day)	15.38%	74.44%

Forty multiparous (in second lactation) Polish Holstein-Friesian cows were selected for the experiment. An additional criterion was the production of at least 2 L of colostrum in the first milking.

The first sample of colostrum was collected up to two hours after calving. Samples were taken individually seven times during the experiment: twice a day on the first and second day after calving, and once a day from three to five days (using the milking machine). The colostrum yield was medium (3–6 kg) for all cows, and foremilk was stripped out of the gland prior to sample collection. Colostrum samples and milk (250 ml) were placed in sterile bottles, preserved with Milkstat CC (Zekar Sp. z o. o., Poland), and transported to the Warsaw University of Life Sciences.

After preliminary analysis of colostrum SCC distribution, cows were divided into two groups based on the SCC of the first sample collected after calving: 1) SCC ≤400,000 cells/ml (range of SCC values: 120,000–400,000 cells/ml); LCC (n = 22), and 2) SCC ≥400,000 cells/ml (range of SCC values: 650,000–1 200,000 cells/ml); GCC (n = 18 cows). It should be noted that the colostrum samples met the microbiological quality requirements for bacterial contamination (≤100,000 CFU/ml).

### Chemical analyses

The microbiological quality of colostrum was determined by Bacto-Scan (Bentley, Warsaw, Poland).

The basic chemical composition of the colostrum, i.e., fat, protein, casein, density, and lactose content, was determined by automated infrared analysis using a Milkoscan FT– 120 analyzer (Foss Electric, Denmark).

Cytological quality (somatic cell count; SCC) was established using a Somacount 150 analyzer (Bentley, Warsaw, Poland).

Concentrations of lactoferrin and lysozyme were determined using an Agilent 1100 Series RP-HPLC (Agilent Technologies, Waldbronn, Germany) according to the methodology described by Puppel et al. [24]. Separations were performed at ambient temperature using solvent gradient on Jupiter column C18 300A (Phenomenex, Torrance, CA, USA). The chromatographic conditions were as follows. Solvent A was acetonitrile (Merck, Darmstadt, Germany), water (Sigma-Aldrich) and trifluoroacetic acid (Sigma-Aldrich) in a ratio of 50:950:1 (v/v/v). Solvent B was acetonitrile, water, and trifluoroacetic acid in a ratio of 950:50:1(v/v/v). The flow rate was 1.2 ml/min and the detection wavelength was 220 nm. The injection volume of final solution was 25 μL. All samples were analyzed in duplicate. The identification of peaks as lactoferrin and lysozyme was confirmed by a comparison with the standards: LF and LZ (Sigma-Aldrich, USA).

Concentrations of immunoglobulins were determined using an Agilent 1100 Series RP-HPLC (Agilent Technologies, Waldbronn, Germany) according to the methodology described by Puppel et al. [24]. Separations were performed using solvent gradient on Jupiter column C18 300A (Phenomenex, Torrance, CA, USA). The chromatographic conditions were as follows. Solvent A was acetonitrile (Merck, Darmstadt, Germany), water (Sigma-Aldrich) and trifluoroacetic acid (Sigma-Aldrich) in a ratio of 10:990:1 (v/v/v). Solvent B was acetonitrile, water, and trifluoroacetic acid in a ratio of 990:10:1(v/v/v). The column was first equilibrated at 25% mobile phase A for 2 min at a 2 mL/min flow rate. The elution was performed as a gradient of mobile phase A, from 25% to 60% over 5 min at 2 mL/min. The detection wavelength was 280 nm. The injection volume of final solution was 25 μL. All samples were analyzed in duplicate. The identification of peaks as immunoglobulins was confirmed by a comparison with the standards of Bovine IgG (Sigma-Aldrich, USA).

Fatty acid methylation was carried out with the *trans-*esterification method PN-EN ISO 5509:2000 [25]. Contents of individual fatty acids were determined in crude fat using an Agilent 7890A GC (Agilent, Waldbronn, Germany) according to Puppel et al. [26]. Each peak was identified using pure methyl ester standards: Supelco 37 Comp. FAME Mix, Lot LB 68887; Methyl linoleate, Lot 094K1497; CLA Conjugated (9Z, 11E), Lot BCBV3726 (Supelco, USA). All samples were analyzed in duplicate.

### Statistical analysis

The experimental data were statistically analyzed by two-way ANOVA according the model presented below using SPSS 23 [27]. To determine differences between means Tukey’s multiple comparison post-hoc test was applied followed by preliminary one-way ANOVA whether there was any evidence that the means of the selected group differed. Data were presented as least squares means with standard error of the mean.

The statistical model was:

$$Y_{ijk}=\mu +A_{i}+B_{j}+\left(A_{i}\times B_{j}\right)+e_{ijk}$$

where: y is the dependent variable, μ is the overall mean, A_i_ is the fixed effect of the colostrum sample (I = 1 − 7), B_j_ is the fixed effect of the SCC level, A_i_ × B_j_ is the interaction between subsequent colostrum sample effect and SCC level, and e_ijk_ is the residual error.

## Results and discussion

Auldist and Hubble [28] report that intramammary infection is quickly followed by an influx of leucocytes into the milk and an increase in the concentration of somatic cells. During the inflammatory process, three mechanisms are responsible for changes in colostrum composition: suppressed synthesis of colostrum components, increased permeability of the blood-colostrum barrier, and increased proteolytic/enzymatic activity [29]. These changes can result in lower concentrations of colostrum components [30], for example disruption of the blood-milk barrier induced by inflammatory processes result in movement of IgG1 from colostrum to serum, thereby lowering colostral IgG1 concentration [21].

**Fig 1:**
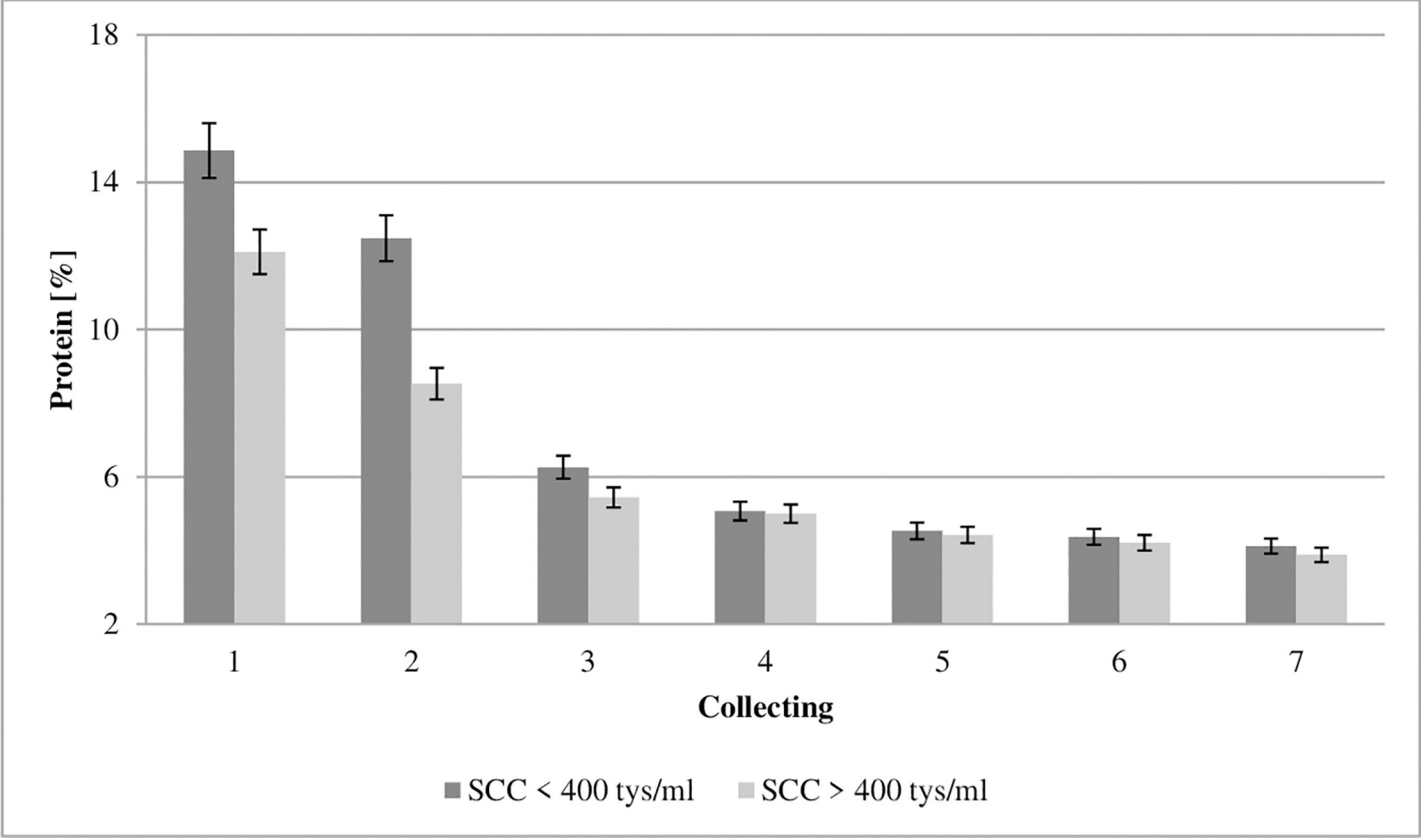
Effect of somatic cell counts on changes in immunoglobulin G concentration of colostrum. SCC–somatic cell counts. Colostrum and milk samples were taken individually from each cow seven times during the experiment: from the first to second day after calving–twice per day, from the third to fifth day after calving–once per day. Data were presented as least squares means with standard error of mean. Statistical differences between SCC groups at P ≤0.01 and collections at P ≤0.01.

Bovine immunoglobulins include IgG (G1 and G2), M, and A, with IgG accounting for 65–90% of total colostral immunoglobulins [31]. Colostral IgG concentration is essential to ensuring adequate passive transfer. Studies have shown that the chemical composition and the immunoglobulin concentration of colostrum varies during the first three days of lactation with breed and age of cow [19,32] and with the system of production [18]. The concentration of IgG in the colostrum from GCC and LCC groups in the first collection was 82.45 and 41.11 g/L, respectively, and decreased successively from day one to day five (Fig 1). Studies have shown that the concentration of immunoglobulin G was significantly influenced by SCC. It should be noted that bacteria can bind free IgG in the intestinal gut or block the uptake and transport of IgG molecules into enterocytes, and consequently impair IgG absorption [33]. Additionally Ferdowsi et al. [20], reported, that colostral IgG concentrations may be insufficient to defining colostral quality, because serum IgG concentrations at parturition showed the linearly increased with colostral SCC, so the cows’ immune system was challenged by the mammary infections leading to elevated IgG synthesis.

**Fig 2:**
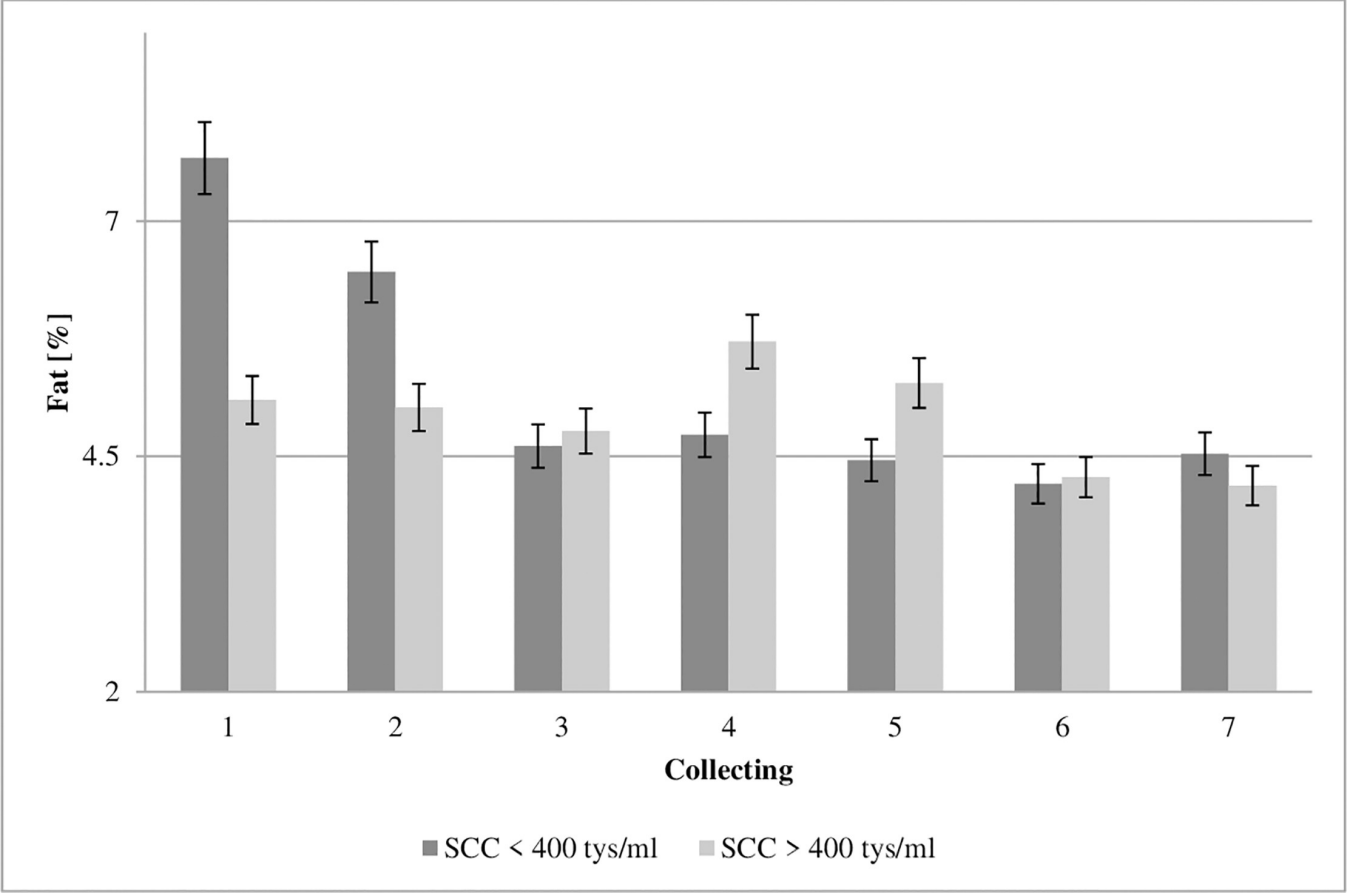
Effect of somatic cell counts on protein concentration of colostrum. SCC–somatic cell counts. Colostrum and milk samples were taken individually from each cow seven times during the experiment: from the first to second day after calving–twice per day, from the third to fifth day after calving–once per day. Data were presented as least squares means with standard error of mean. Statistical differences between SCC groups at P≤0.01 and collections at P≤0.01.

**Fig 3:**
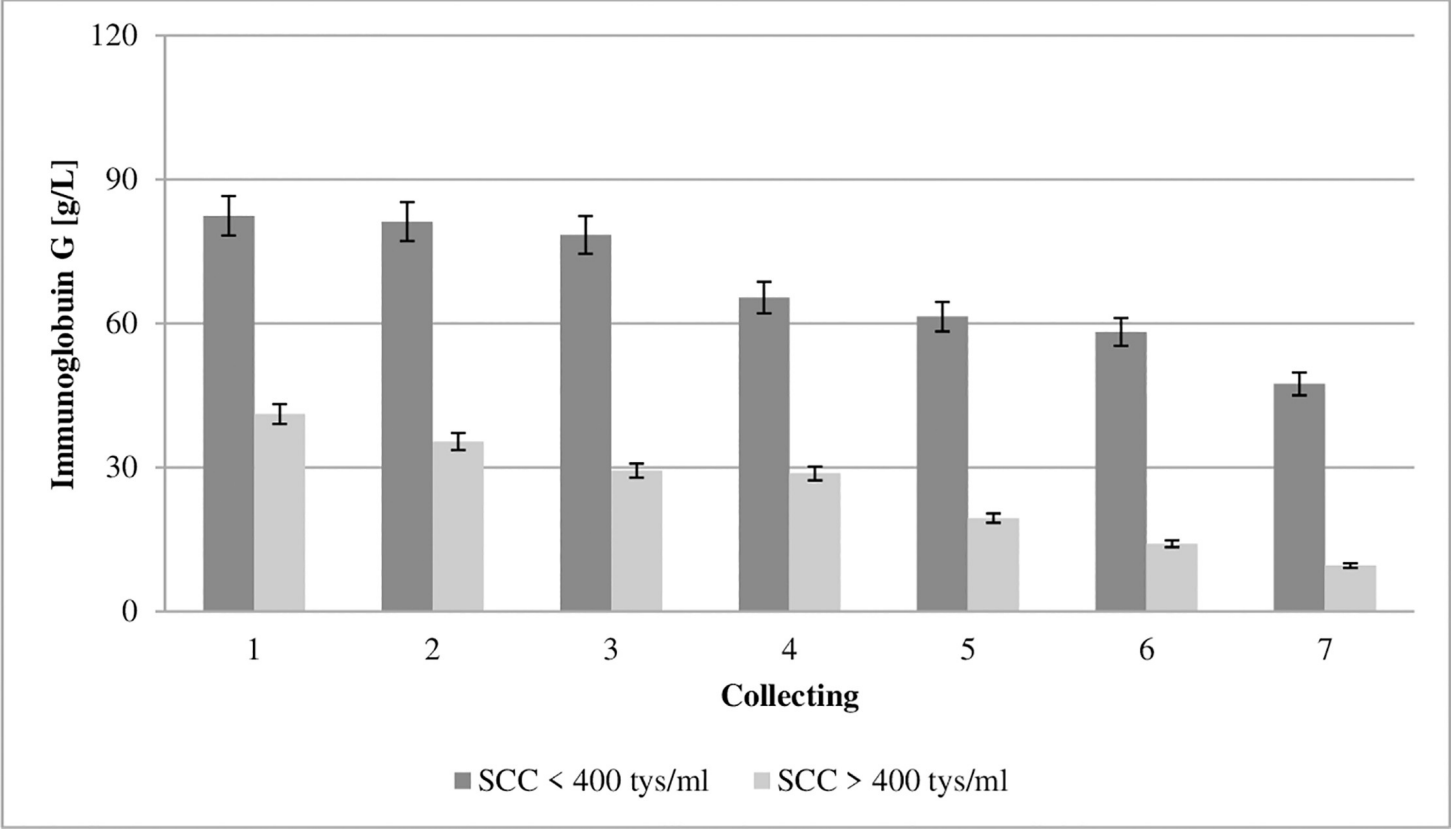
Effect of somatic cell counts on fat concentration of colostrum. SCC–somatic cell counts. Colostrum and milk samples were taken individually from each cow seven times during the experiment: from the first to second day after calving–twice per day, from the third to fifth day after calving–once per day. Data were presented as least squares means with standard error of mean. Statistical differences between SCC groups at P≤0.01 and collections at P≤0.01.

**Fig 4:**
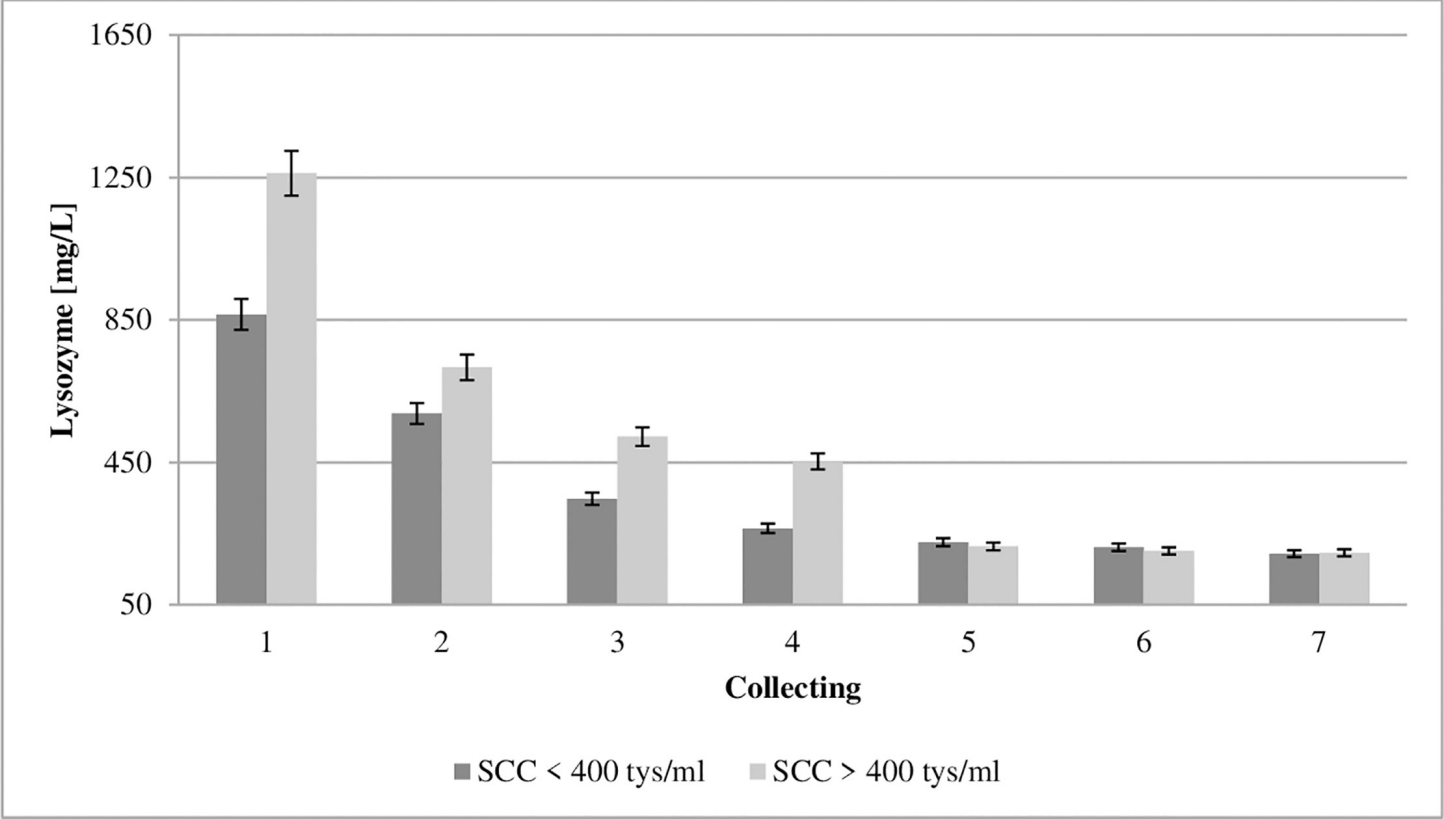
Effect of somatic cell counts on changes in lysozyme concentration of colostrum. SCC–somatic cell counts. Colostrum and milk samples were taken individually from each cow seven times during the experiment: from the first to second day after calving–twice per day, from the third to fifth day after calving–once per day. Data were presented as least squares means with standard error of mean. Statistical differences between SCC groups at P ≤0.01 and collections at P ≤0.01.

**Fig 5:**
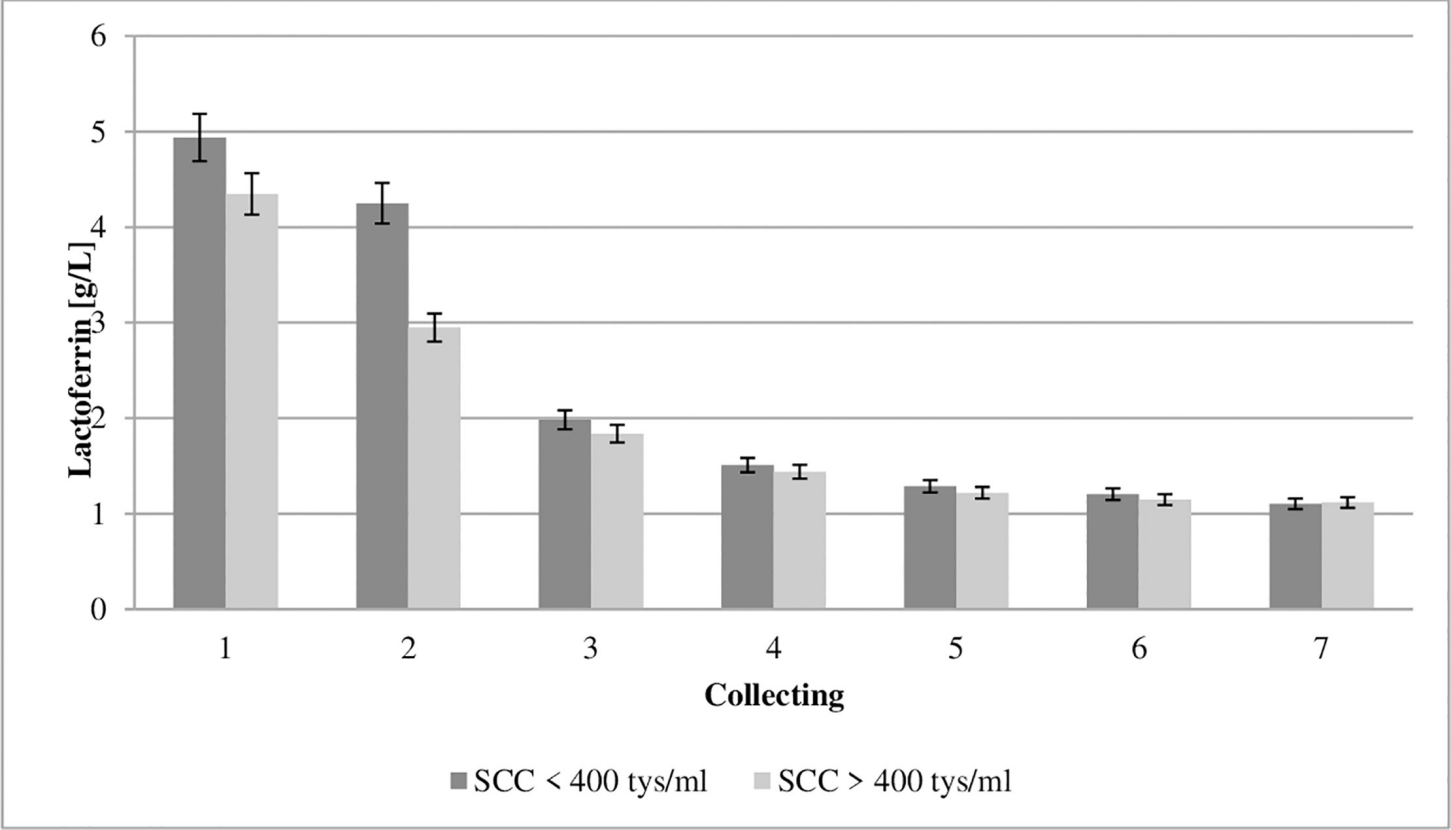
Effect of somatic cell counts on changes in lactoferrin concentration of colostrum. SCC–somatic cell counts. Colostrum and milk samples were taken individually from each cow seven times during the experiment: from the first to second day after calving–twice per day, from the third to fifth day after calving–once per day. Data were presented as least squares means with standard error of mean. Statistical differences between SCC groups at P ≤0.01 and collections at P ≤0.01.

**Fig 6:**
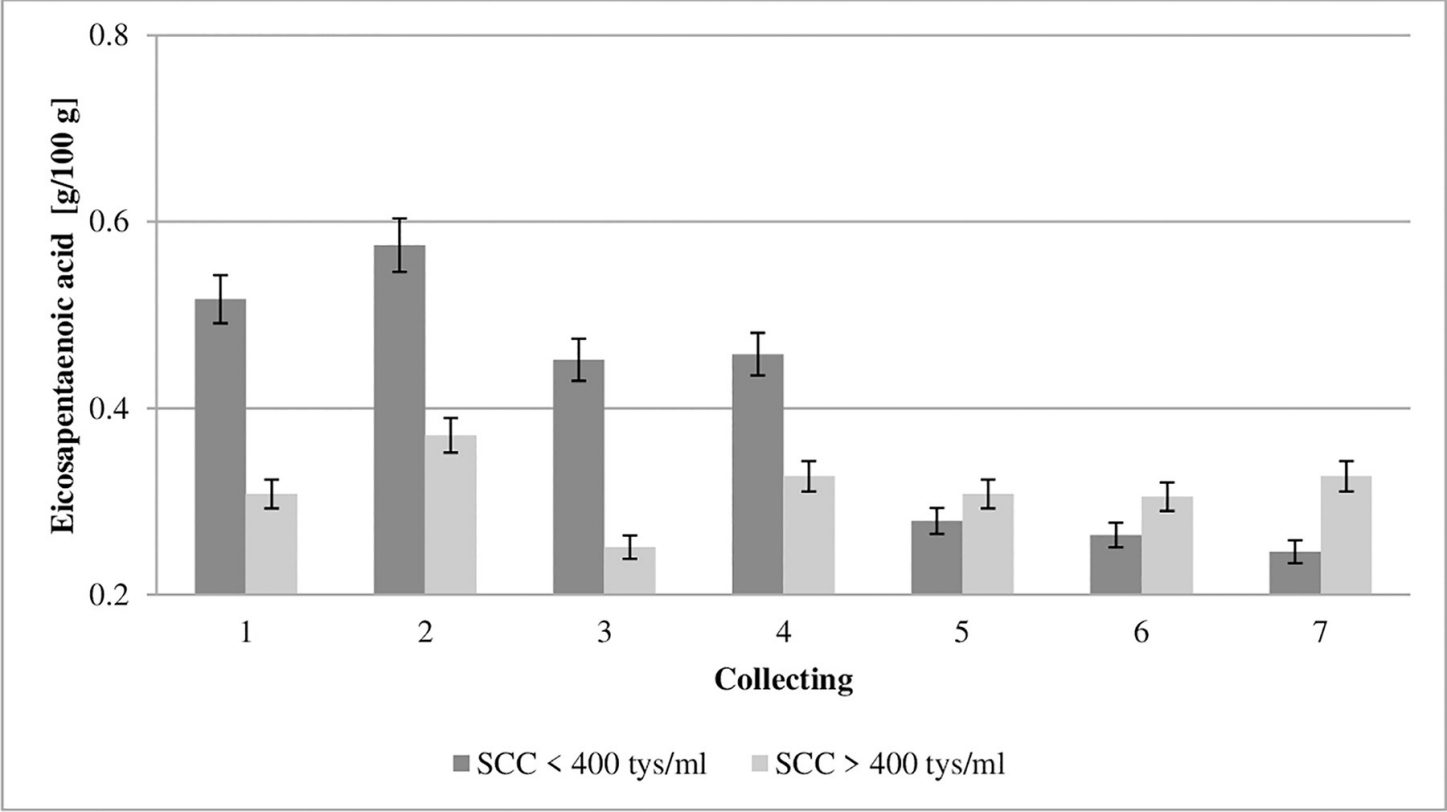
Effect of somatic cell counts on changes in eicosapentaenoic acid content of colostrum. SCC–somatic cell counts. Colostrum and milk samples were taken individually from each cow seven times during the experiment: from the first to second day after calving–twice per day, from the third to fifth day after calving–once per day. Data were presented as least squares means with standard error of mean. Statistical differences between SCC groups at P ≤0.01 and collections at P ≤0.01.

**Fig 7:**
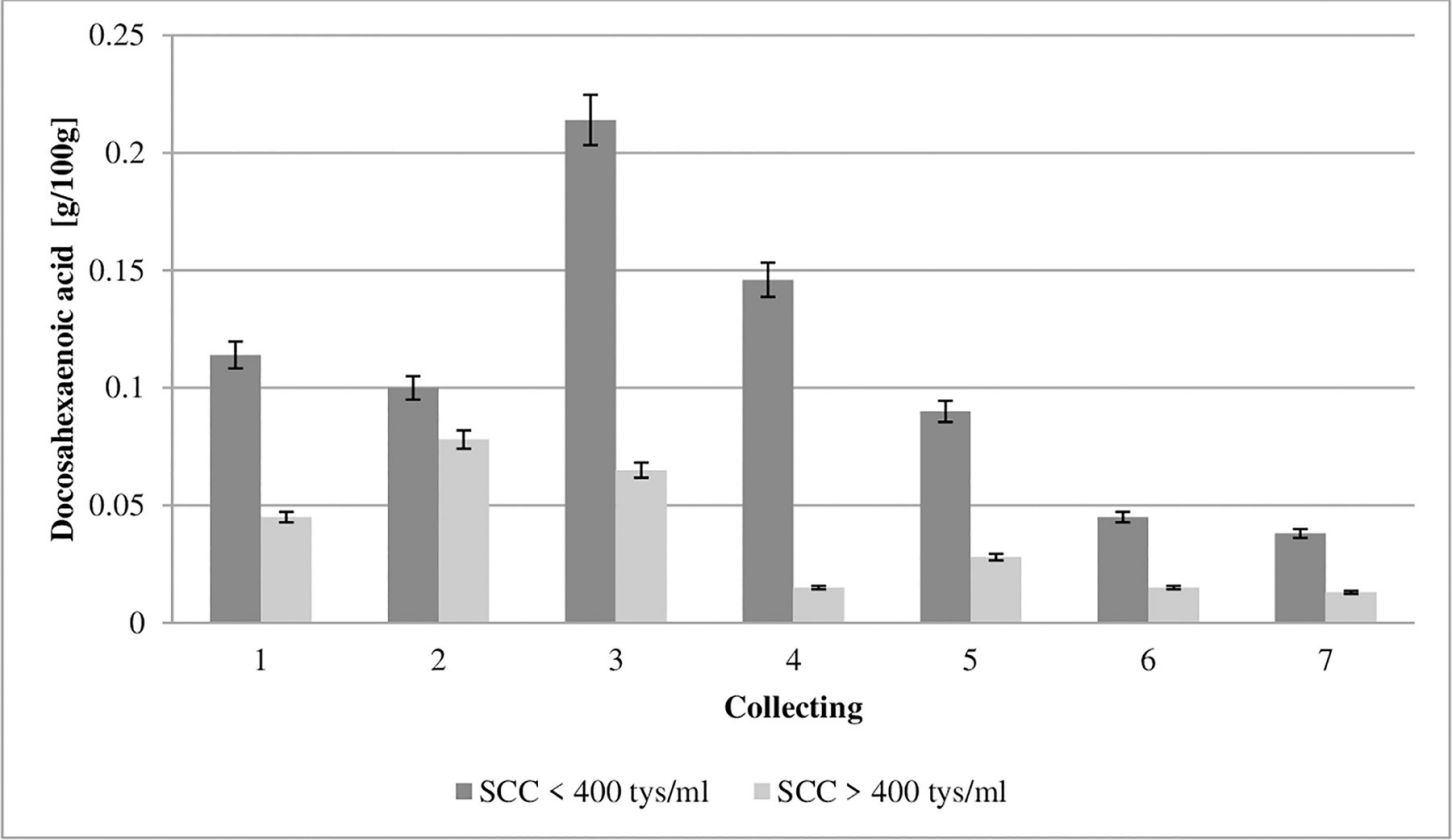
Effect of somatic cell counts on changes in docosahexaenoic acid content of colostrum. SCC–somatic cell counts. Colostrum and milk samples were taken individually from each cow seven times during the experiment: from the first to second day after calving–twice per day, from the third to fifth day after calving–once per day. Data were presented as least squares means with standard error of mean. Statistical differences between SCC groups at P ≤0.01 and collections at P ≤0.01.

**Fig 8:**
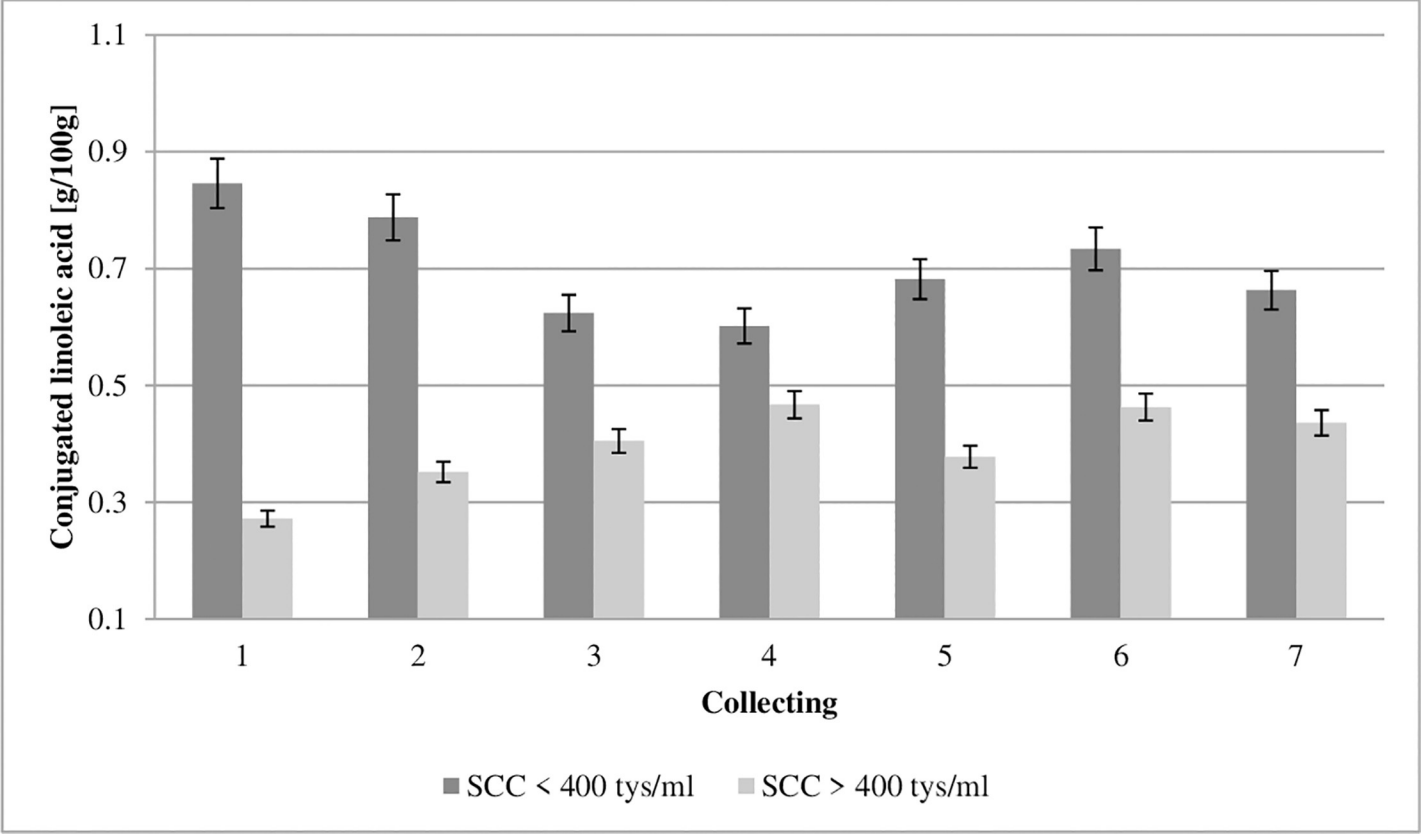
Effect of somatic cell counts on changes in conjugated linoleic acid content of colostrum. SCC–somatic cell counts. Colostrum and milk samples were taken individually from each cow seven times during the experiment: from the first to second day after calving–twice per day, from the third to fifth day after calving–once per day. Data were presented as least squares means with standard error of mean. Statistical differences between SCC groups at P ≤0.01 and collections at P ≤0.01.

The quality of colostrum varies, with that variability being determined by animal and environmental factors [19,34,35]. The composition of colostrum changes by over time [23]. Its biological value drops, as presented in Figs 1–8. Between the second and fifth days of lactation, organic compounds and electrolytes start fluctuating within the udder secretion, after which their composition stabilizes [1].

Figs 2 and 3 show that the protein and fat concentration of colostrum during the first seven collections differed between cows with low and high SCC at the first collection. The biological properties of proteins are connected with facilitating nutrient assimilation, whereas peptides (which are derivatives of proteins) affect both the growth and differentiation of neonatal tissues [23]. This study found a protein concentration at the level of 14.86% in first milking colostrum with ≤400,000 cells/ml SCC (GCC) and 12.11% in the samples with ≥400,000 cells/ml SCC (LCC) (Fig 2). Our data shows that the concentration of protein was significantly influenced by SCC.

The fat in the colostrum is an important element of a calf’s diet by providing the energy necessary to maintain body heat, and also by acting as a precursor for the synthesis of certain enzymes and hormones [1,31,35]. The calf is born with a small amount of lipids in its body weight, because lipids represent barely 3% [35,36]. In the first collection of colostrum, a fat concentration of 7.67% was found in GCC and 5.11% in LCC (Fig 3). Significantly lower fat concentration in the LCC group suggests that de novo synthesis of fatty acids and acylation with glycerol of long-chain fatty acids may be impaired in infected mammary cells [37]. Our data shows that the concentration of fat was significantly influenced by SCC.

There is also a different group of proteins which are very significant in terms of the colostrum’s bacteriostatic and germicidal properties, that includes the non-specific antibiotic agents lysozyme and lactoferrin. Lysozyme, also known as muramidase, is a hydrolytic enzyme [38], which owes its bactericidal capacity to the fact that it catalyzes the decomposition of murein (dissolves the polysaccharide-peptide complex), which is also known as peptidoglycan [39]. Its monomer also shows certain bactericidal properties [40]. Lysozyme is a germicide effective in almost all body fluids. The only resistance has been shown in the case of lactic acid and propionic acid bacteria [41]. The concentration of lysozyme in the colostrum from the GCC and LCC groups in the first collection was 865.52 and 1261.99 mg/L, respectively, and dropped successively (Fig 4). Due to its resistance to digestive proteases, it may remain active while passing through the small intestine [42]. Paulík et al. [43] described that the concentration of lysozyme and immunoglobulins of the IgG and IgM class in colostrum had the opposite trend in the first and second milkings after calving. The increase in the lysozyme level is associated with a fall in the concentration of immunoglobulins, which was confirmed in the present study.

The lactoferrin (LF) content in bovine colostrum ranges between 0.34 and 4.96 g/L [18,44,45]. Due to its iron-binding capability, it exerts a bacteriostatic effect and inhibits the development of bacteria [46]. The concentration of LF in the colostrum from the GCC and LCC groups in the first collection was 4.93 and 4.34 g/L, respectively, and decreased successively (Fig 5). Robblee et al. [47] proved LF to be a beneficial dietary supplement for newborn calves because it improved health. In addition, Habring et al. [48] showed that LF administered as a treatment to calves with diarrhea significantly reduced mortality.

Cyclooxygenases and lipoxygenases from PUFAs produce both pro-inflammatory molecules (prostaglandins and leukotrienes), and anti-inflammatory molecules (lipoxins and resolvins) [9]. Therefore, PUFAs affect the development of inflammation, and the balance between these antagonistic compounds can determine the emergence of the pathological process [49]. Eicosapentaenoic acid (EPA, C20:5 n-3) and docosahexaenoic acid (DHA, C22:6 n-3) inhibit proinflammatory cytokines and macrophage migration, as well as the production of HMGB1 protein and TNF-α by T cells and other cells, and therefore can function as endogenous anti-inflammatory molecules [50]. The ability of EPA and DHA to suppress the production of pro-inflammatory cytokines and induce their anti-inflammatory effects results indirectly from their ability to increase mRNA PPAR-γ and protein activity [9]. The present study showed an almost double EPA and DHA level in the first collection in the colostrum from the GCC group compared to the values obtained in LCC group (Figs 6 and 7). Our study shows that the concentrations of EPA and DHA were significantly influenced by SCC.

Kisza and Botura [51] demonstrated that mastitic milk contained more short- and medium-chain fatty acids and a reduced amount of unsaturated fatty acids than milk from healthy animals. Therefore, it may be concluded that colostrum of good cytological quality significantly affects the natural defense mechanisms of the calf because the above-mentioned acids exhibit the anti-inflammatory activity–they reduce the synthesis of proinflammatory cytokines and also the production of enzymes involved in the formation of proinflammatory eicosanoids, and enhance the synthesis of resolvin and protactin.

Conjugated linoleic acid (CLA, C18:2 *cis*-9 *trans*-11) inhibits prostaglandin-E2 synthesis [52] and metabolism of PGF2α, leukotriene-B4, and leukotriene-C4 derived from arachidonic acid [53,54]. In addition, eicosanoid receptors control the release of messengers that are essential for such processes as cell proliferation, differentiation, and apoptosis [55]. In addition, CLA plays a key role in lipid metabolism, including in the oxidative cell system [56]. The concentration of CLA in the colostrum from the GCC and LCC groups in the first collection was 0.8476 and 0.272 g/100 g of fat, respectively (Fig 8). The study revealed an almost three-times higher concentration of CLA in the GCC, than in the LCC group. Our study shows that the concentration of CLA was significantly influenced by SCC.

## Conclusion

In this study, the concentration of IgG, Lz, Lf, CLA, EPA and DHA in colostrum were significantly influenced by SCC. In first milking colostrum, the IgG concentration was increased 2-fold and the CLA was increased three-fold in the low SCC group (≤400,000 cells/ml) compared with the LCC group (≥400,000 cells/ml). It should be emphasized that the quality of colostrum varies, with this variability being significantly influenced by the level of somatic cells. Therefore, it can be concluded that SCC can be used as non-invasive indicator of colostrum quality.

## Supporting information

S1 File The results obtained for individual cows. (PDF)
