# Supplementary material for: Use of somatic cell count as an indicator of colostrum quality
Source: PLoS One. 2020 Aug 11;15(8):e0237615. doi: 10.1371/journal.pone.0237615 (PMC7418990; doi:10.1371/journal.pone.0237615)
Supplement: S1 File — (PDF) [file pone.0237615.s001.pdf]

lab nr	Protein [	Fat [%]	SNF [%]	Lactose	LF [g/L]	ALA [g/L	BLG [g/L	c4:0
1	13,330	6,540	17,180	2,340	4,825	3,950	7,644	1,402
8	13,060	8,490	16,980	2,620	4,213	2,069	7,724	1,005
15	7,000	4,270	9,210	1,500	3,050	1,498	9,707	0,859
22	13,010	8,280	16,710	2,390	4,588	2,253	14,601	1,438
29	10,210	2,400	14,850	3,310	3,650	1,793	11,617	0,957
36	8,750	7,990	12,820	2,930	2,988	1,467	9,508	0,819
43	15,430	4,130	18,840	1,890	5,763	2,830	12,657	0,700
50	15,190	3,840	19,190	2,430	5,575	2,738	12,060	0,854
71	10,670	6,650	14,590	2,500	3,675	1,805	11,696	0,955
78	14,180	4,360	18,240	2,600	5,150	2,529	16,391	1,331
92	11,050	3,540	15,550	2,830	3,975	1,952	12,651	1,167
99	13,160	2,790	17,040	2,500	4,975	2,443	15,834	2,190
106	14,890	4,350	18,400	2,000	5,550	2,726	17,664	0,840
117	13,586	6,665	17,510	2,385	4,918	4,025	7,791	1,429
124	13,311	8,653	17,306	2,670	4,293	2,109	7,872	1,025
131	7,134	4,352	9,387	1,529	3,109	1,527	9,893	0,875
135	13,260	8,439	17,031	2,436	4,675	2,296	14,881	1,465
142	10,406	2,446	15,135	3,373	3,720	1,827	11,840	0,975
149	8,918	8,143	13,066	2,986	3,045	1,495	9,691	0,835
156	15,726	4,209	19,201	1,926	5,873	2,884	12,900	0,713
163	15,481	3,914	19,558	2,477	5,682	2,791	12,291	0,871
165	10,875	6,778	14,870	2,548	3,745	1,840	11,921	0,974
172	14,452	4,444	18,590	2,650	5,249	2,578	16,705	1,356
179	17,708	3,939	23,018	3,174	6,390	3,139	20,339	0,000
186	13,645	4,371	19,202	3,495	4,909	2,411	15,622	1,441
193	16,251	3,445	21,042	3,087	6,143	3,017	19,553	2,704
200	18,387	5,372	22,721	2,470	6,853	3,366	21,812	1,037
211	16,776	8,231	21,622	2,945	6,072	4,971	9,620	1,765
218	16,437	10,685	21,370	3,297	5,302	2,604	9,721	1,265
225	8,810	5,374	11,591	1,888	3,839	1,885	12,217	1,081
229	16,374	10,421	21,030	3,008	5,774	2,836	18,375	1,810
236	12,850	3,020	18,689	4,166	4,594	2,256	14,620	1,204
243	11,012	10,056	16,134	3,688	3,760	1,847	11,967	1,031
250	19,419	5,198	23,711	2,379	7,252	3,562	15,929	0,880
257	19,117	4,833	24,151	3,058	7,016	3,446	15,178	1,075
259	13,429	8,369	18,362	3,146	4,625	2,272	14,720	1,203
266	17,846	5,487	22,956	3,272	6,481	3,183	20,629	1,675
2	11,260	8,400	15,290	2,580	3,950	1,940	12,572	1,292
9	6,130	3,540	10,970	3,730	1,925	0,945	6,127	1,032
16	7,650	9,060	12,150	3,430	2,200	1,081	7,002	0,981
23	7,760	7,780	11,940	2,960	2,675	1,314	8,514	1,325
30	6,130	3,060	11,030	3,750	2,050	1,007	6,525	1,093
37	6,680	6,730	11,050	3,260	2,213	1,087	7,042	0,841
44	10,320	2,760	14,310	2,660	3,863	1,897	6,610	0,799
72	3,320	2,040	5,950	2,070	1,788	0,878	5,689	1,092
79	10,430	3,990	14,800	3,160	3,663	1,799	11,657	1,226
86	11,680	5,570	15,950	2,860	3,913	1,922	12,452	1,482
93	7,350	5,020	12,180	3,420	2,425	1,191	7,718	1,121
100	9,570	1,920	14,070	3,140	3,525	1,731	11,219	1,818

107	10,320	3,510	14,610	3,030	3,688	1,811	11,736	0,862
113	11,820	7,210	16,110	2,720	4,063	1,995	12,930	0,717
118	11,476	8,561	15,583	2,629	4,026	1,977	12,813	1,317
125	6,248	3,608	11,180	3,802	1,962	0,964	6,244	1,052
132	7,797	9,234	12,383	3,496	2,242	1,101	7,136	1,000
136	7,909	7,929	12,169	3,017	2,726	1,339	8,677	1,350
143	6,248	3,119	11,242	3,822	2,089	1,026	6,650	1,114
150	6,808	6,859	11,262	3,323	2,255	1,107	7,177	0,857
157	10,518	2,813	14,584	2,711	3,937	1,933	6,737	0,815
166	3,384	2,079	6,064	2,110	1,822	0,895	5,798	1,113
173	10,630	4,067	15,084	3,221	3,733	1,833	11,880	1,249
180	14,423	6,878	19,696	3,532	4,831	2,373	15,377	1,829
187	9,076	6,199	15,041	4,223	2,995	1,471	9,531	1,385
194	11,818	2,371	17,374	3,877	4,353	2,138	13,854	2,245
201	12,744	4,334	18,041	3,742	4,554	2,236	14,492	1,065
207	14,596	8,903	19,894	3,359	5,017	2,464	15,966	0,886
212	14,171	10,572	19,243	3,247	4,971	2,442	15,822	1,626
219	7,715	4,455	13,806	4,694	2,423	1,190	7,711	1,299
226	9,628	11,402	15,291	4,317	2,769	1,360	8,812	1,235
230	9,766	9,791	15,027	3,725	3,367	1,653	10,715	1,667
237	7,715	3,851	13,882	4,720	2,580	1,267	8,211	1,376
244	8,407	8,470	13,907	4,103	2,785	1,368	8,862	1,058
251	12,988	3,474	18,010	3,348	4,861	2,387	8,319	1,006
260	4,178	2,567	7,488	2,605	2,250	1,105	7,160	1,374
267	13,127	5,022	18,626	3,977	4,609	2,264	14,670	1,543
3	6,980	6,390	11,670	3,520	2,263	1,111	7,201	1,365
10	2,410	1,710	5,240	2,340	1,375	0,675	4,376	1,420
17	5,520	7,610	10,350	3,720	1,550	0,761	4,933	1,315
24	5,380	6,250	10,080	3,490	1,675	0,823	5,331	1,399
31	4,930	3,580	9,930	3,900	1,563	0,767	4,973	1,466
38	4,340	4,020	9,210	3,830	1,350	0,663	4,297	1,157
45	7,360	4,230	11,910	3,350	2,488	1,222	7,917	1,072
73	2,770	1,270	5,580	2,210	1,600	0,786	5,092	1,464
80	6,070	3,370	10,940	3,810	1,975	0,970	6,286	1,295
87	6,830	4,010	11,860	3,770	2,088	1,025	6,644	1,365
94	5,530	4,410	10,650	3,770	1,738	0,853	5,530	1,503
101	7,120	1,590	12,190	3,640	2,488	1,222	7,917	1,675
108	2,660	1,610	5,490	2,360	1,488	0,731	4,734	1,186
114	6,940	5,670	11,980	3,770	2,113	1,038	6,723	0,820
119	7,114	6,513	11,894	3,588	2,306	1,133	7,339	1,391
126	2,456	1,743	5,341	2,385	1,401	0,688	4,460	1,447
133	5,626	7,756	10,549	3,791	1,580	0,776	5,028	1,340
137	5,483	6,370	10,273	3,557	1,707	0,838	5,433	1,426
144	5,025	3,649	10,120	3,975	1,592	0,782	5,068	1,494
151	4,423	4,097	9,387	3,903	1,376	0,676	4,379	1,179
158	7,501	4,311	12,138	3,414	2,535	1,245	8,069	1,092
167	2,823	1,294	5,687	2,252	1,631	0,801	5,190	1,492
174	7,496	4,161	13,509	4,705	2,439	1,198	7,762	1,599
181	8,434	4,952	14,645	4,655	2,578	1,266	8,204	1,685
188	6,829	5,446	13,151	4,655	2,146	1,054	6,829	1,856

195	8,792	1,963	15,053	4,495	3,072	1,509	9,776	2,068
202	3,285	1,988	6,779	2,914	1,837	0,902	5,846	1,465
208	8,570	7,002	14,794	4,655	2,609	1,281	8,302	1,012
213	8,785	8,042	14,687	4,430	2,847	1,398	9,063	1,718
220	3,033	2,152	6,595	2,945	1,730	0,850	5,508	1,787
227	6,947	9,577	13,026	4,682	1,951	0,958	6,209	1,655
231	6,771	7,866	12,686	4,392	2,108	1,035	6,709	1,761
238	6,205	4,506	12,497	4,908	1,966	0,966	6,259	1,845
245	5,462	5,059	11,591	4,820	1,699	0,834	5,408	1,456
252	9,263	5,324	14,989	4,216	3,131	1,538	9,964	1,349
261	3,486	1,598	7,023	2,781	2,014	0,989	6,409	1,842
268	9,256	5,139	16,682	5,810	3,012	1,479	9,585	1,975
4	4,940	6,010	9,890	3,920	1,425	0,700	4,535	1,400
11	4,580	6,840	9,630	4,090	1,100	0,540	3,501	0,900
18	4,830	3,490	10,010	3,950	1,475	0,724	4,694	0,795
25	4,620	5,030	9,540	3,720	1,450	0,712	4,615	1,436
32	4,320	2,280	9,230	3,880	1,438	0,706	4,575	0,886
39	4,290	5,820	9,160	3,920	1,188	0,583	3,779	0,733
46	5,840	4,710	10,580	3,640	1,838	0,902	5,848	0,647
74	4,500	6,400	9,000	3,530	1,375	0,675	4,376	0,884
81	5,380	3,880	10,410	3,960	1,650	0,810	5,251	1,329
88	5,740	5,560	10,890	3,950	1,513	0,743	4,814	1,442
95	4,830	3,650	10,150	4,030	1,488	0,731	4,734	0,908
102	5,020	3,090	10,130	3,910	1,613	0,792	5,132	1,769
109	4,210	4,270	9,400	4,210	1,138	0,559	3,620	0,752
115	5,110	5,590	10,380	4,130	1,388	0,681	4,416	2,687
120	5,035	6,125	10,080	3,995	1,452	0,713	4,622	1,427
127	4,668	6,971	9,815	4,168	1,121	0,551	3,568	0,917
134	4,923	3,557	10,202	4,026	1,503	0,738	4,785	0,810
138	4,709	5,126	9,723	3,791	1,478	0,726	4,703	1,463
145	4,403	2,324	9,407	3,954	1,465	0,720	4,663	0,903
152	4,372	5,932	9,336	3,995	1,210	0,594	3,852	0,747
159	5,952	4,800	10,783	3,710	1,873	0,920	5,960	0,660
168	4,586	6,523	9,173	3,598	1,401	0,688	4,460	0,901
175	6,644	4,791	12,855	4,890	2,038	1,001	6,485	1,641
182	7,088	6,866	13,448	4,878	1,868	0,917	5,944	1,781
189	5,964	4,507	12,534	4,976	1,837	0,902	5,846	1,122
196	6,199	3,816	12,509	4,828	1,991	0,978	6,337	2,185
203	5,199	5,273	11,608	5,199	1,405	0,690	4,471	0,928
209	6,310	6,903	12,818	5,100	1,713	0,841	5,453	3,318
214	6,217	7,564	12,447	4,933	1,793	0,881	5,708	1,762
221	5,764	8,608	12,120	5,147	1,384	0,680	4,406	1,132
228	6,079	4,392	12,598	4,971	1,856	0,912	5,908	1,000
232	5,814	6,330	12,006	4,682	1,825	0,896	5,808	1,807
239	5,437	2,869	11,616	4,883	1,809	0,889	5,758	1,115
246	5,399	7,325	11,528	4,933	1,495	0,734	4,757	0,922
253	7,350	5,928	13,315	4,581	2,313	1,136	7,360	0,815
262	5,663	8,055	11,327	4,443	1,730	0,850	5,508	1,113
269	8,204	5,916	15,874	6,038	2,516	1,236	8,008	2,026
5	3,610	3,620	8,920	4,280	0,950	0,467	3,024	1,575

12	4,610	7,420	9,850	4,140	1,088	0,534	3,461	1,901
26	4,480	4,450	9,540	3,800	1,400	0,688	4,456	1,615
33	4,460	3,950	9,830	4,230	1,275	0,626	4,058	1,679
40	4,190	6,830	9,180	4,020	1,063	0,522	3,382	1,549
47	4,850	1,730	10,010	4,030	1,563	0,767	4,973	1,773
75	4,310	5,350	9,310	3,910	1,263	0,620	4,018	2,421
82	5,040	5,370	10,090	3,990	1,438	0,706	4,575	1,495
89	4,880	5,240	10,350	4,220	1,288	0,632	4,098	1,479
96	4,340	7,390	9,400	3,980	1,138	0,559	3,620	1,722
103	4,530	4,030	9,820	4,190	1,313	0,645	4,177	1,815
110	4,140	3,490	9,490	4,270	1,150	0,565	3,660	1,588
116	4,410	5,200	9,880	4,370	1,100	0,540	3,501	2,230
121	3,679	3,689	9,091	4,362	0,968	0,476	3,082	1,605
128	4,698	7,562	10,039	4,219	1,108	0,544	3,528	1,937
139	4,566	4,535	9,723	3,873	1,427	0,701	4,541	1,646
146	4,546	4,026	10,019	4,311	1,299	0,638	4,136	1,711
153	4,270	6,961	9,356	4,097	1,083	0,532	3,446	1,578
160	4,943	1,763	10,202	4,107	1,592	0,782	5,068	1,807
169	4,393	5,453	9,489	3,985	1,287	0,632	4,095	2,468
176	6,224	6,631	12,460	4,927	1,775	0,872	5,650	1,846
183	6,026	6,471	12,781	5,211	1,590	0,781	5,060	1,827
190	5,359	9,126	11,608	4,915	1,405	0,690	4,471	2,126
197	5,594	4,976	12,126	5,174	1,621	0,796	5,158	2,241
204	5,112	4,310	11,719	5,273	1,420	0,697	4,520	1,961
210	5,446	6,421	12,200	5,396	1,358	0,667	4,323	2,754
215	4,543	4,556	11,226	5,387	1,196	0,587	3,805	1,982
222	5,802	9,338	12,397	5,210	1,369	0,672	4,356	2,392
233	5,638	5,601	12,006	4,782	1,762	0,865	5,608	2,033
240	5,613	4,971	12,371	5,324	1,605	0,788	5,107	2,113
247	5,273	8,596	11,553	5,059	1,337	0,657	4,256	1,949
254	6,104	2,177	12,598	5,072	1,966	0,966	6,259	2,231
263	5,424	6,733	11,717	4,921	1,589	0,780	5,057	3,047
270	7,685	8,189	15,386	6,084	2,192	1,077	6,976	2,279
6	3,530	3,530	9,020	4,420	0,888	0,436	2,825	2,150
13	4,540	5,320	10,020	4,380	1,100	0,540	3,501	1,864
27	4,430	4,300	9,700	4,090	1,300	0,638	4,138	2,205
34	4,200	3,210	9,720	4,390	1,175	0,577	3,740	1,647
41	4,050	3,000	9,410	4,330	1,138	0,559	3,620	1,519
48	4,160	2,680	9,670	4,450	1,125	0,553	3,581	1,739
76	4,320	4,960	9,560	4,090	1,238	0,608	3,939	2,375
83	4,870	3,660	10,210	4,110	1,438	0,706	4,575	2,040
90	4,540	4,860	10,190	4,330	1,188	0,583	3,779	1,664
97	4,290	4,560	9,760	4,260	1,188	0,583	3,779	1,689
104	4,520	3,830	10,100	4,420	1,238	0,608	3,939	2,042
111	4,120	3,110	9,470	4,330	1,113	0,546	3,541	1,558
122	3,598	3,598	9,193	4,505	0,905	0,444	2,879	2,192
129	4,627	5,422	10,212	4,464	1,121	0,551	3,568	1,900
140	4,515	4,382	9,886	4,168	1,325	0,651	4,217	2,247
147	4,281	3,272	9,906	4,474	1,198	0,588	3,811	1,678
154	4,128	3,058	9,591	4,413	1,159	0,569	3,690	1,548

161	4,240	2,731	9,855	4,535	1,147	0,563	3,649	1,772
170	4,403	5,055	9,743	4,168	1,261	0,619	4,014	2,420
177	6,014	4,520	12,608	5,075	1,775	0,872	5,650	2,520
184	5,606	6,001	12,583	5,347	1,466	0,720	4,667	2,055
191	5,298	5,631	12,052	5,260	1,466	0,720	4,667	2,085
198	5,582	4,729	12,472	5,458	1,528	0,751	4,864	2,521
205	5,088	3,840	11,694	5,347	1,374	0,675	4,372	1,924
216	4,443	4,443	11,352	5,563	1,117	0,549	3,555	2,706
223	5,714	6,695	12,611	5,512	1,384	0,680	4,406	2,346
234	5,575	5,412	12,208	5,147	1,636	0,804	5,207	2,775
241	5,286	4,040	12,233	5,525	1,479	0,726	4,707	2,073
248	5,097	3,776	11,843	5,449	1,432	0,703	4,556	1,912
255	5,236	3,373	12,170	5,601	1,416	0,695	4,506	2,188
264	5,437	6,242	12,032	5,147	1,557	0,765	4,957	2,989
271	7,426	5,581	15,569	6,267	2,192	1,077	6,976	3,111
7	3,610	3,810	9,170	4,470	0,875	0,430	2,785	2,073
14	2,270	2,330	5,160	2,400	1,300	0,638	4,138	2,012
28	4,060	4,090	9,520	4,230	1,138	0,559	3,620	2,126
35	4,140	3,140	9,760	4,460	1,138	0,559	3,620	1,777
42	3,810	2,970	9,300	4,470	1,025	0,503	3,262	1,640
49	4,170	4,740	9,360	4,180	1,125	0,553	3,581	1,877
70	4,270	4,590	9,730	4,090	1,225	0,602	3,899	1,746
77	3,770	4,680	9,020	4,250	1,000	0,491	3,183	2,563
84	4,230	3,730	9,690	4,260	1,175	0,577	3,740	1,967
91	4,250	7,250	9,490	4,060	1,100	0,540	3,501	2,272
98	4,150	6,820	9,470	4,210	1,050	0,516	3,342	1,823
105	4,310	3,300	9,900	4,480	1,175	0,577	3,740	2,787
112	4,250	3,570	9,850	4,460	1,125	0,553	3,581	1,681
123	3,679	3,883	9,346	4,556	0,892	0,438	2,838	2,113
130	2,314	2,375	5,259	2,446	1,325	0,651	4,217	2,051
141	4,138	4,168	9,703	4,311	1,159	0,569	3,690	2,166
148	4,219	3,200	9,947	4,546	1,159	0,569	3,690	1,812
155	3,883	3,027	9,478	4,556	1,045	0,513	3,325	1,671
162	4,250	4,831	9,540	4,260	1,147	0,563	3,649	1,913
164	4,352	4,678	9,917	4,168	1,248	0,613	3,974	1,780
171	3,842	4,770	9,193	4,332	1,019	0,501	3,244	2,612
178	5,223	4,606	11,966	5,260	1,451	0,713	4,618	2,429
185	5,248	8,953	11,719	5,014	1,358	0,667	4,323	2,805
192	5,125	8,422	11,694	5,199	1,297	0,637	4,127	2,251
199	5,322	4,075	12,225	5,532	1,451	0,713	4,618	3,442
206	5,248	4,408	12,163	5,507	1,389	0,682	4,421	2,076
217	4,543	4,795	11,541	5,626	1,101	0,541	3,505	2,609
224	2,857	2,932	6,494	3,020	1,636	0,804	5,207	2,533
235	5,110	5,147	11,981	5,324	1,432	0,703	4,556	2,675
242	5,210	3,952	12,283	5,613	1,432	0,703	4,556	2,237
249	4,795	3,738	11,704	5,626	1,290	0,634	4,106	2,063
256	5,248	5,965	11,780	5,261	1,416	0,695	4,506	2,362
258	5,374	5,777	12,246	5,147	1,542	0,757	4,907	2,197
265	4,745	5,890	11,352	5,349	1,259	0,618	4,006	3,226
272	6,450	5,688	14,776	6,496	1,792	0,880	5,702	2,999

c6:0	c8:0	c8:1	c12:0	c12:1	c14:0	c14:1	c15:0	c15:1
0,705	1,688	0,077	3,101	0,059	11,130	0,816	0,852	0,160
0,523	1,490	0,063	2,579	0,045	13,229	1,308	0,709	0,117
0,447	1,272	0,054	2,202	0,039	11,299	1,117	0,605	0,100
0,723	1,731	0,079	3,180	0,061	11,413	0,837	0,873	0,164
0,498	1,418	0,060	2,455	0,043	12,593	1,245	0,675	0,111
0,426	1,214	0,052	2,101	0,037	10,778	1,065	0,577	0,095
0,364	1,037	0,044	1,794	0,031	9,206	0,910	0,493	0,081
0,445	1,266	0,054	2,192	0,038	11,245	1,111	0,602	0,099
0,497	1,416	0,060	2,451	0,043	12,574	1,243	0,674	0,111
0,669	1,602	0,073	2,942	0,056	10,562	0,774	0,808	0,152
0,607	1,730	0,074	2,994	0,052	9,170	1,518	0,823	0,136
0,978	1,930	0,083	2,220	0,128	6,756	0,428	0,829	0,268
0,437	1,245	0,053	2,154	0,038	11,053	1,093	0,592	0,098
0,718	1,720	0,078	3,160	0,061	11,344	0,832	0,868	0,163
0,533	1,518	0,065	2,628	0,046	13,483	1,333	0,722	0,119
0,455	1,297	0,055	2,245	0,039	11,516	1,138	0,617	0,102
0,737	1,764	0,080	3,241	0,062	11,632	0,853	0,890	0,167
0,507	1,445	0,061	2,502	0,044	12,835	1,269	0,688	0,114
0,434	1,237	0,053	2,141	0,037	10,985	1,086	0,589	0,097
0,371	1,057	0,045	1,829	0,032	9,383	0,927	0,503	0,083
0,453	1,291	0,055	2,234	0,039	11,460	1,133	0,614	0,101
0,507	1,443	0,061	2,498	0,044	12,815	1,267	0,687	0,113
0,682	1,633	0,074	2,999	0,057	10,764	0,789	0,824	0,154
0,000	0,000	0,000	0,000	0,000	0,000	0,000	0,000	0,000
0,750	2,136	0,091	3,697	0,065	11,323	1,875	1,016	0,168
1,208	2,383	0,103	2,741	0,157	8,342	0,529	1,024	0,332
0,540	1,537	0,065	2,660	0,047	13,649	1,349	0,731	0,121
0,887	2,125	0,096	3,902	0,075	14,008	1,027	1,072	0,201
0,658	1,875	0,080	3,245	0,057	16,649	1,646	0,892	0,147
0,562	1,601	0,068	2,772	0,049	14,220	1,406	0,762	0,126
0,910	2,178	0,099	4,002	0,077	14,363	1,053	1,099	0,206
0,627	1,785	0,076	3,089	0,054	15,849	1,567	0,849	0,140
0,536	1,528	0,065	2,644	0,046	13,565	1,341	0,727	0,120
0,458	1,305	0,056	2,258	0,040	11,586	1,145	0,621	0,102
0,559	1,594	0,068	2,758	0,048	14,152	1,399	0,758	0,125
0,626	1,782	0,076	3,085	0,054	15,825	1,564	0,848	0,140
0,842	2,016	0,091	3,703	0,071	13,292	0,975	1,017	0,191
0,693	1,686	0,073	2,963	0,058	10,535	0,724	0,836	0,157
0,645	1,690	0,061	2,421	0,058	11,511	0,819	0,759	0,154
0,597	1,418	0,046	2,197	0,057	10,470	0,468	0,729	0,139
0,711	1,728	0,075	3,038	0,059	10,803	0,742	0,857	0,161
0,666	1,581	0,052	2,449	0,064	11,669	0,521	0,812	0,155
0,525	1,377	0,050	1,973	0,047	9,379	0,667	0,618	0,126
0,487	1,155	0,038	1,790	0,046	8,531	0,381	0,594	0,113
0,665	1,578	0,052	2,445	0,064	11,652	0,520	0,811	0,154
0,658	1,600	0,070	2,811	0,055	9,997	0,687	0,793	0,149
0,745	1,783	0,081	3,276	0,063	11,759	0,862	0,900	0,169
0,683	1,621	0,053	2,511	0,065	11,966	0,534	0,833	0,159
0,914	2,188	0,099	3,135	0,077	11,807	1,058	1,104	0,207

0,539	1,412	0,051	2,023	0,049	9,618	0,684	0,634	0,129
0,373	1,063	0,045	1,840	0,032	9,441	0,933	0,506	0,084
0,706	1,718	0,075	3,019	0,059	10,737	0,738	0,852	0,160
0,657	1,722	0,062	2,468	0,059	11,732	0,834	0,774	0,157
0,609	1,445	0,047	2,239	0,058	10,671	0,477	0,743	0,141
0,724	1,762	0,077	3,096	0,060	11,010	0,757	0,874	0,164
0,679	1,611	0,053	2,496	0,065	11,893	0,531	0,828	0,158
0,535	1,403	0,051	2,011	0,048	9,559	0,680	0,630	0,128
0,496	1,178	0,038	1,824	0,047	8,694	0,388	0,605	0,115
0,678	1,608	0,053	2,492	0,065	11,875	0,530	0,827	0,157
0,670	1,630	0,071	2,865	0,056	10,189	0,700	0,809	0,152
0,920	2,202	0,100	4,045	0,078	14,521	1,065	1,111	0,208
0,843	2,002	0,065	3,101	0,081	14,777	0,660	1,029	0,196
1,128	2,702	0,123	3,872	0,095	14,580	1,306	1,364	0,256
0,665	1,744	0,063	2,498	0,060	11,877	0,845	0,783	0,159
0,461	1,313	0,056	2,272	0,040	11,658	1,152	0,625	0,103
0,872	2,121	0,092	3,729	0,073	13,259	0,911	1,052	0,198
0,812	2,127	0,077	3,047	0,073	14,488	1,030	0,955	0,194
0,752	1,785	0,058	2,765	0,072	13,177	0,589	0,917	0,175
0,894	2,175	0,095	3,823	0,075	13,596	0,934	1,079	0,203
0,838	1,989	0,065	3,082	0,080	14,686	0,656	1,022	0,195
0,661	1,733	0,063	2,483	0,060	11,804	0,840	0,778	0,158
0,613	1,454	0,048	2,253	0,059	10,736	0,480	0,747	0,142
0,837	1,986	0,065	3,077	0,080	14,664	0,655	1,021	0,194
0,828	2,013	0,088	3,538	0,069	12,582	0,865	0,999	0,188
0,828	2,032	0,094	3,165	0,065	9,589	0,617	0,217	0,861
0,868	2,151	0,080	2,599	0,068	10,208	0,570	0,787	0,170
0,800	1,871	0,058	2,351	0,061	9,206	0,385	0,681	0,148
0,849	2,083	0,097	3,246	0,066	9,833	0,633	0,223	0,882
0,892	2,086	0,065	2,620	0,068	10,260	0,429	0,759	0,165
0,707	1,752	0,065	2,117	0,055	8,317	0,464	0,641	0,139
0,652	1,525	0,047	1,915	0,050	7,500	0,313	0,555	0,121
0,891	2,083	0,065	2,616	0,068	10,245	0,428	0,758	0,165
0,786	1,928	0,089	3,004	0,062	9,099	0,586	0,206	0,817
0,732	1,781	0,077	3,130	0,061	11,130	0,765	0,883	0,166
0,915	2,139	0,067	2,687	0,070	10,521	0,440	0,779	0,169
0,898	2,185	0,095	2,956	0,075	12,346	0,938	1,084	0,204
0,725	1,797	0,067	2,171	0,057	8,529	0,476	0,657	0,142
0,499	1,185	0,039	1,836	0,048	8,748	0,391	0,609	0,116
0,844	2,071	0,096	3,226	0,066	9,773	0,629	0,221	0,877
0,885	2,192	0,081	2,648	0,069	10,404	0,581	0,802	0,173
0,816	1,907	0,059	2,396	0,063	9,382	0,392	0,694	0,151
0,866	2,123	0,098	3,308	0,068	10,021	0,645	0,227	0,899
0,909	2,126	0,066	2,670	0,070	10,457	0,437	0,774	0,168
0,721	1,786	0,066	2,158	0,056	8,477	0,473	0,653	0,141
0,665	1,554	0,048	1,952	0,051	7,644	0,319	0,566	0,123
0,908	2,123	0,066	2,666	0,070	10,441	0,436	0,773	0,168
0,971	2,381	0,110	3,709	0,076	11,236	0,724	0,254	1,008
0,904	2,199	0,096	3,865	0,075	13,744	0,944	1,091	0,205
1,130	2,641	0,082	3,318	0,087	12,992	0,543	0,961	0,209

1,109	2,698	0,117	3,651	0,093	15,246	1,159	1,338	0,252
0,896	2,219	0,082	2,681	0,070	10,532	0,588	0,812	0,175
0,616	1,463	0,048	2,267	0,059	10,802	0,482	0,752	0,143
1,043	2,557	0,118	3,984	0,082	12,068	0,777	0,273	1,083
1,093	2,707	0,100	3,270	0,086	12,847	0,717	0,990	0,214
1,007	2,355	0,073	2,959	0,077	11,586	0,484	0,857	0,186
1,069	2,622	0,121	4,085	0,084	12,375	0,797	0,280	1,111
1,123	2,625	0,082	3,297	0,086	12,913	0,540	0,956	0,208
0,890	2,205	0,082	2,665	0,070	10,467	0,584	0,807	0,174
0,821	1,919	0,060	2,411	0,063	9,440	0,395	0,699	0,152
1,121	2,621	0,082	3,292	0,086	12,893	0,539	0,954	0,207
1,199	2,940	0,136	4,580	0,094	13,875	0,893	0,314	1,245
0,813	1,933	0,099	2,909	0,034	8,685	0,555	0,870	0,182
0,987	2,406	0,101	2,868	0,078	10,756	0,512	0,867	0,188
0,872	2,125	0,089	2,533	0,069	9,501	0,452	0,765	0,166
0,834	1,982	0,102	2,982	0,034	8,906	0,570	0,892	0,187
0,972	2,369	0,099	2,823	0,077	10,589	0,504	0,853	0,185
0,805	1,960	0,082	2,337	0,063	8,763	0,417	0,706	0,153
0,711	1,732	0,072	2,064	0,056	7,741	0,369	0,624	0,135
0,971	2,365	0,099	2,819	0,076	10,573	0,503	0,852	0,184
0,772	1,834	0,094	2,760	0,032	8,242	0,527	0,825	0,173
0,875	2,147	0,099	3,344	0,068	10,131	0,652	0,229	0,909
0,997	2,429	0,102	2,895	0,079	10,858	0,517	0,875	0,189
1,074	2,634	0,122	3,219	0,084	12,430	0,800	0,281	1,116
0,825	2,010	0,084	2,396	0,065	8,986	0,428	0,724	0,157
1,200	2,368	0,102	2,723	0,156	8,289	0,526	1,017	0,329
0,829	1,970	0,101	2,964	0,034	8,852	0,566	0,886	0,186
1,006	2,452	0,103	2,923	0,079	10,962	0,522	0,883	0,191
0,889	2,166	0,091	2,582	0,070	9,683	0,461	0,780	0,169
0,850	2,020	0,104	3,040	0,035	9,077	0,580	0,909	0,190
0,991	2,414	0,101	2,877	0,078	10,792	0,514	0,869	0,188
0,820	1,998	0,084	2,381	0,065	8,931	0,425	0,720	0,156
0,724	1,765	0,074	2,103	0,057	7,889	0,376	0,636	0,138
0,989	2,410	0,101	2,873	0,078	10,776	0,513	0,868	0,188
0,953	2,265	0,116	3,408	0,039	10,177	0,651	1,019	0,213
1,081	2,651	0,123	4,129	0,085	12,510	0,806	0,283	1,123
1,231	2,999	0,126	3,575	0,097	13,409	0,638	1,080	0,234
1,326	3,252	0,151	3,975	0,104	15,350	0,988	0,347	1,378
1,019	2,482	0,104	2,959	0,080	11,097	0,528	0,894	0,194
1,482	2,924	0,126	3,363	0,193	10,236	0,649	1,256	0,407
1,024	2,433	0,125	3,661	0,042	10,931	0,699	1,095	0,229
1,243	3,028	0,127	3,609	0,098	13,536	0,645	1,091	0,236
1,098	2,675	0,112	3,188	0,086	11,957	0,569	0,963	0,209
1,050	2,495	0,128	3,754	0,043	11,209	0,717	1,122	0,235
1,223	2,981	0,125	3,553	0,096	13,326	0,635	1,074	0,233
1,013	2,467	0,103	2,941	0,080	11,029	0,525	0,889	0,192
0,894	2,179	0,091	2,598	0,070	9,742	0,464	0,785	0,170
1,222	2,977	0,125	3,548	0,096	13,306	0,634	1,072	0,232
1,177	2,797	0,143	4,209	0,048	12,568	0,804	1,258	0,263
0,966	2,042	0,111	2,582	0,061	7,327	0,682	0,904	0,219

1,264	3,223	0,145	3,857	0,093	11,659	0,431	0,905	0,251
0,991	2,094	0,114	2,648	0,062	7,513	0,699	0,927	0,225
1,116	2,847	0,128	3,407	0,083	10,298	0,381	0,799	0,222
1,030	2,626	0,118	3,142	0,076	9,499	0,352	0,737	0,205
1,178	3,005	0,135	3,597	0,087	10,873	0,402	0,844	0,234
1,610	4,105	0,184	4,913	0,119	11,314	0,550	1,153	0,320
0,917	1,938	0,105	2,450	0,058	6,953	0,647	0,858	0,208
0,859	2,042	0,105	3,073	0,035	9,176	0,587	0,919	0,192
1,145	2,919	0,131	3,494	0,085	10,561	0,391	0,820	0,227
1,054	2,506	0,128	2,886	0,043	11,259	0,720	1,127	0,236
1,056	2,692	0,121	3,222	0,078	9,741	0,361	0,756	0,210
1,121	2,685	0,122	3,164	0,094	11,150	1,298	1,355	0,254
0,985	2,082	0,113	2,632	0,062	7,468	0,695	0,921	0,223
1,288	3,284	0,147	3,931	0,095	11,882	0,440	0,922	0,256
1,010	2,134	0,116	2,698	0,063	7,657	0,713	0,945	0,229
1,138	2,901	0,130	3,472	0,084	10,496	0,388	0,815	0,226
1,049	2,676	0,120	3,203	0,078	9,681	0,358	0,752	0,209
1,201	3,063	0,138	3,666	0,089	11,081	0,410	0,860	0,239
1,640	4,184	0,188	5,007	0,121	11,531	0,560	1,175	0,326
1,133	2,393	0,130	3,026	0,071	8,586	0,799	1,059	0,257
1,061	2,522	0,129	3,795	0,044	11,331	0,725	1,135	0,238
1,413	3,605	0,162	4,314	0,105	13,041	0,483	1,012	0,281
1,302	3,095	0,159	3,564	0,054	13,903	0,889	1,392	0,291
1,304	3,325	0,149	3,979	0,096	12,029	0,445	0,934	0,259
1,385	3,316	0,150	3,907	0,117	13,768	1,603	1,673	0,314
1,216	2,570	0,140	3,250	0,076	9,221	0,858	1,138	0,276
1,590	4,056	0,182	4,854	0,118	14,673	0,543	1,139	0,316
1,247	2,636	0,143	3,332	0,078	9,456	0,880	1,167	0,283
1,405	3,583	0,161	4,288	0,104	12,961	0,480	1,006	0,279
1,296	3,304	0,148	3,955	0,096	11,955	0,442	0,928	0,257
1,483	3,782	0,170	4,527	0,110	13,684	0,506	1,062	0,295
2,026	5,166	0,232	6,183	0,150	14,239	0,692	1,451	0,403
1,398	2,955	0,160	3,736	0,088	10,602	0,987	1,308	0,317
0,943	1,886	0,086	2,202	0,095	6,501	0,256	0,838	0,234
1,182	2,888	0,157	3,422	0,111	11,705	0,422	0,962	0,277
0,967	1,934	0,088	2,258	0,098	6,666	0,262	0,859	0,239
1,044	2,551	0,138	3,023	0,098	10,339	0,373	0,850	0,244
0,963	2,353	0,128	2,788	0,090	9,536	0,344	0,784	0,225
1,103	2,694	0,146	3,192	0,103	10,916	0,394	0,897	0,258
1,506	3,679	0,200	4,359	0,141	11,373	0,538	1,225	0,352
0,895	1,790	0,082	2,089	0,091	6,169	0,243	0,795	0,222
1,021	2,158	0,117	2,728	0,064	7,741	0,721	0,955	0,231
1,071	2,616	0,142	3,100	0,100	10,602	0,383	0,871	0,250
1,253	2,648	0,144	3,347	0,079	9,498	0,884	1,172	0,284
0,988	2,413	0,131	2,859	0,092	9,779	0,353	0,804	0,231
0,961	1,922	0,088	2,244	0,097	6,626	0,261	0,854	0,238
1,205	2,944	0,160	3,488	0,113	11,929	0,431	0,980	0,282
0,986	1,971	0,090	2,301	0,100	6,794	0,267	0,876	0,244
1,064	2,600	0,141	3,081	0,099	10,537	0,380	0,866	0,249
0,982	2,398	0,130	2,842	0,092	9,719	0,351	0,799	0,230

1,124	2,745	0,149	3,253	0,105	11,125	0,402	0,914	0,263
1,535	3,750	0,203	4,443	0,143	11,591	0,548	1,249	0,359
1,105	2,210	0,101	2,580	0,112	7,618	0,300	0,982	0,274
1,261	2,665	0,145	3,369	0,079	9,559	0,890	1,179	0,286
1,322	3,231	0,175	3,828	0,124	13,092	0,473	1,076	0,309
1,547	3,269	0,177	4,133	0,097	11,729	1,092	1,447	0,351
1,220	2,980	0,162	3,531	0,114	12,076	0,436	0,992	0,285
1,187	2,374	0,109	2,771	0,120	8,182	0,322	1,055	0,294
1,488	3,635	0,197	4,307	0,139	14,731	0,532	1,211	0,348
1,217	2,434	0,111	2,842	0,123	8,390	0,330	1,082	0,301
1,314	3,211	0,174	3,805	0,123	13,012	0,470	1,069	0,307
1,212	2,962	0,161	3,509	0,113	12,002	0,433	0,986	0,284
1,388	3,390	0,184	4,017	0,130	13,738	0,496	1,129	0,325
1,895	4,630	0,251	5,487	0,177	14,313	0,677	1,542	0,443
1,365	2,729	0,125	3,186	0,138	9,407	0,370	1,213	0,338
0,926	1,826	0,079	2,101	0,121	6,394	0,406	0,785	0,254
1,355	3,163	0,167	3,564	0,124	10,850	0,335	0,900	0,265
0,949	1,873	0,081	2,154	0,124	6,557	0,416	0,804	0,261
1,197	2,794	0,147	3,148	0,110	9,584	0,296	0,795	0,234
1,104	2,577	0,136	2,904	0,101	8,840	0,273	0,733	0,216
1,263	2,950	0,155	3,324	0,116	10,119	0,313	0,840	0,247
0,780	1,538	0,067	1,770	0,102	5,386	0,342	0,661	0,214
1,726	4,029	0,212	4,540	0,158	10,285	0,427	1,147	0,338
0,879	1,733	0,075	1,994	0,115	6,068	0,385	0,744	0,241
0,997	1,993	0,091	2,326	0,101	6,868	0,270	0,885	0,247
1,227	2,865	0,151	3,228	0,113	9,829	0,304	0,815	0,240
1,223	2,445	0,112	2,854	0,124	8,427	0,331	1,086	0,303
1,132	2,643	0,139	2,978	0,104	9,066	0,280	0,752	0,222
0,944	1,862	0,080	2,141	0,123	6,517	0,413	0,800	0,259
1,381	3,224	0,170	3,632	0,127	11,058	0,342	0,918	0,270
0,968	1,909	0,083	2,196	0,126	6,683	0,424	0,820	0,266
1,220	2,848	0,150	3,208	0,112	9,768	0,302	0,810	0,239
1,125	2,627	0,138	2,959	0,103	9,010	0,278	0,748	0,220
1,288	3,006	0,158	3,387	0,118	10,313	0,319	0,856	0,252
0,795	1,568	0,068	1,804	0,104	5,489	0,348	0,674	0,218
1,759	4,106	0,216	4,627	0,161	10,482	0,435	1,169	0,344
1,085	2,140	0,093	2,462	0,141	7,493	0,475	0,919	0,298
1,231	2,461	0,113	2,873	0,125	8,481	0,334	1,093	0,305
1,515	3,538	0,186	3,986	0,139	12,137	0,375	1,007	0,297
1,510	3,019	0,138	3,525	0,153	10,406	0,409	1,342	0,374
1,398	3,264	0,172	3,677	0,128	11,195	0,346	0,929	0,274
1,165	2,299	0,099	2,644	0,152	8,048	0,510	0,987	0,320
1,705	3,981	0,210	4,485	0,156	13,656	0,422	1,133	0,334
1,195	2,357	0,102	2,711	0,156	8,252	0,523	1,012	0,328
1,506	3,516	0,185	3,962	0,138	12,062	0,373	1,001	0,295
1,389	3,244	0,171	3,654	0,127	11,126	0,344	0,923	0,272
1,590	3,713	0,195	4,183	0,146	12,735	0,393	1,057	0,311
0,981	1,936	0,084	2,227	0,128	6,779	0,430	0,832	0,269
2,172	5,071	0,267	5,713	0,199	12,944	0,537	1,443	0,425
1,340	2,643	0,114	3,040	0,175	9,252	0,587	1,135	0,368

c16:0	c16:1	c17:0	c17:1	c18:0	c18:1 tra	c18:1 cis	c18:2 n6	c18:3 n6
32,591	1,141	0,331	0,577	5,845	1,018	24,117	1,965	0,105
42,390	3,197	0,208	0,149	3,661	0,538	17,574	1,371	0,046
36,207	2,731	0,178	0,128	3,127	0,460	15,010	1,171	0,039
33,419	1,170	0,340	0,592	5,994	1,044	24,729	2,015	0,108
40,353	3,043	0,198	0,142	3,485	0,513	16,729	1,305	0,044
34,538	2,605	0,170	0,122	2,982	0,439	14,318	1,117	0,037
29,500	2,225	0,145	0,104	2,547	0,375	12,230	0,954	0,032
36,032	2,717	0,177	0,127	3,112	0,458	14,938	1,165	0,039
40,293	3,039	0,198	0,142	3,479	0,512	16,704	1,303	0,044
30,927	1,083	0,314	0,548	5,547	0,966	22,885	1,865	0,099
31,533	1,944	0,242	0,173	4,250	0,625	20,403	1,591	0,053
26,939	0,877	0,468	0,126	13,405	1,772	26,864	1,684	0,124
35,418	2,671	0,174	0,125	3,058	0,450	14,683	1,145	0,038
33,216	1,163	0,338	0,588	5,957	1,038	24,579	2,003	0,107
43,203	3,258	0,212	0,152	3,731	0,549	17,911	1,397	0,047
36,901	2,783	0,181	0,130	3,187	0,469	15,298	1,193	0,040
34,060	1,193	0,346	0,603	6,108	1,064	25,204	2,054	0,110
41,127	3,102	0,202	0,145	3,551	0,522	17,050	1,330	0,045
35,200	2,655	0,173	0,124	3,040	0,447	14,593	1,138	0,038
30,065	2,267	0,148	0,106	2,596	0,382	12,464	0,972	0,033
36,723	2,770	0,180	0,129	3,171	0,467	15,224	1,187	0,040
41,066	3,097	0,202	0,145	3,546	0,522	17,025	1,328	0,044
31,520	1,104	0,320	0,558	5,653	0,985	23,324	1,901	0,101
0,000	0,000	0,000	0,000	0,000	0,000	0,000	0,000	0,000
38,939	2,400	0,298	0,214	5,248	0,772	25,195	1,965	0,066
33,266	1,083	0,577	0,156	16,553	2,188	33,173	2,080	0,153
43,736	3,298	0,215	0,154	3,777	0,556	18,132	1,414	0,047
41,017	1,436	0,417	0,726	7,356	1,282	30,352	2,474	0,132
53,350	4,024	0,262	0,188	4,607	0,678	22,117	1,725	0,058
45,567	3,437	0,224	0,161	3,935	0,579	18,891	1,473	0,049
42,059	1,473	0,428	0,745	7,543	1,314	31,123	2,536	0,135
50,786	3,830	0,249	0,179	4,386	0,645	21,054	1,642	0,055
43,467	3,278	0,213	0,153	3,754	0,552	18,020	1,405	0,047
37,126	2,800	0,182	0,131	3,206	0,472	15,391	1,200	0,040
45,348	3,420	0,223	0,160	3,916	0,576	18,800	1,466	0,049
50,710	3,824	0,249	0,179	4,379	0,644	21,023	1,640	0,055
38,922	1,363	0,396	0,689	6,981	1,216	28,802	2,347	0,125
32,015	1,087	0,327	0,116	6,324	1,034	23,795	1,903	0,084
36,899	2,303	0,294	0,156	7,381	1,030	24,465	1,579	0,105
35,419	1,454	0,281	0,136	7,016	1,027	28,857	1,845	0,074
32,828	1,115	0,335	0,119	6,484	1,060	24,400	1,951	0,087
36,474	1,621	0,314	0,151	7,819	1,145	27,162	2,056	0,083
30,063	1,876	0,239	0,127	6,014	0,839	19,933	1,287	0,085
28,858	1,185	0,229	0,111	5,716	0,837	23,512	1,503	0,060
34,112	1,618	0,313	0,151	7,808	1,143	27,114	2,053	0,082
30,380	1,032	0,310	0,110	6,001	0,981	22,580	1,806	0,080
34,433	1,206	0,350	0,610	6,175	1,076	25,479	2,076	0,111
31,640	1,662	0,322	0,155	8,019	1,174	28,981	2,109	0,085
28,102	1,479	0,429	0,748	7,577	1,320	22,422	2,548	0,136

30,829	1,924	0,245	0,130	6,167	0,861	20,441	1,320	0,088
30,251	2,281	0,149	0,107	2,612	0,384	12,541	0,978	0,033
32,629	1,108	0,333	0,119	6,445	1,054	24,252	1,939	0,086
37,606	2,347	0,299	0,159	7,523	1,050	24,935	1,610	0,107
36,099	1,482	0,287	0,138	7,151	1,047	29,411	1,880	0,075
33,458	1,136	0,342	0,122	6,609	1,080	24,868	1,989	0,088
37,173	1,652	0,320	0,154	7,969	1,167	27,683	2,096	0,084
30,640	1,912	0,244	0,129	6,129	0,856	20,316	1,312	0,087
29,412	1,208	0,234	0,113	5,826	0,853	23,963	1,532	0,061
34,766	1,649	0,319	0,154	7,958	1,165	27,634	2,093	0,084
30,962	1,051	0,316	0,113	6,116	1,000	23,013	1,840	0,082
42,519	1,489	0,432	0,753	7,626	1,329	31,463	2,564	0,137
39,071	2,052	0,397	0,192	9,902	1,450	35,788	2,604	0,104
34,702	1,827	0,530	0,924	9,357	1,630	27,687	3,146	0,168
38,070	2,376	0,303	0,160	7,615	1,063	25,242	1,630	0,108
37,356	2,817	0,183	0,132	3,226	0,475	15,487	1,208	0,040
40,292	1,368	0,412	0,147	7,959	1,301	29,947	2,395	0,106
46,438	2,898	0,370	0,196	9,289	1,297	30,791	1,988	0,132
44,576	1,830	0,354	0,171	8,830	1,293	36,318	2,322	0,093
41,316	1,403	0,422	0,150	8,161	1,334	30,708	2,456	0,109
45,904	2,040	0,395	0,191	9,841	1,441	34,184	2,588	0,104
37,836	2,361	0,301	0,159	7,569	1,056	25,087	1,620	0,108
36,319	1,491	0,289	0,139	7,194	1,054	29,590	1,892	0,076
42,931	2,037	0,394	0,190	9,826	1,439	34,124	2,584	0,104
38,234	1,298	0,391	0,139	7,552	1,235	28,418	2,273	0,101
31,410	1,061	0,356	0,225	6,548	1,216	26,719	1,656	0,109
32,030	1,781	0,327	0,195	8,553	1,297	28,053	1,305	0,111
32,449	1,534	0,313	0,133	8,400	1,259	9,944	1,545	0,081
32,208	1,088	0,365	0,231	6,714	1,246	18,557	1,698	0,112
32,029	1,709	0,349	0,148	9,362	1,404	28,644	1,722	0,091
26,097	1,451	0,267	0,159	6,968	1,057	22,856	1,063	0,090
26,438	1,250	0,255	0,108	6,844	1,026	29,712	1,259	0,066
33,458	1,707	0,349	0,148	9,348	1,402	22,901	1,720	0,091
29,806	1,007	0,338	0,214	6,213	1,153	25,354	1,571	0,104
33,824	1,149	0,345	0,123	6,681	1,092	25,140	2,010	0,089
28,245	1,753	0,358	0,152	9,600	1,439	28,679	1,766	0,093
27,355	1,409	0,424	0,151	8,197	1,340	22,005	2,467	0,109
26,762	1,488	0,274	0,163	7,146	1,084	23,439	1,090	0,093
29,593	1,215	0,235	0,114	5,862	0,858	24,111	1,542	0,062
32,013	1,081	0,363	0,230	6,674	1,239	27,232	1,688	0,111
32,645	1,815	0,334	0,198	8,717	1,322	28,591	1,330	0,113
33,071	1,563	0,319	0,135	8,561	1,284	10,135	1,575	0,083
32,826	1,109	0,372	0,235	6,843	1,270	18,913	1,731	0,114
32,643	1,742	0,356	0,151	9,542	1,431	29,193	1,755	0,092
26,598	1,479	0,272	0,162	7,102	1,077	23,295	1,084	0,092
26,945	1,274	0,260	0,110	6,975	1,046	30,282	1,283	0,068
34,100	1,739	0,355	0,150	9,527	1,428	23,340	1,753	0,092
36,806	1,243	0,417	0,264	7,673	1,424	31,309	1,940	0,128
41,767	1,418	0,427	0,152	8,250	1,349	31,044	2,483	0,110
34,878	2,164	0,442	0,187	11,855	1,777	35,415	2,181	0,115

33,780	1,740	0,523	0,186	10,123	1,655	27,173	3,046	0,135
33,047	1,837	0,338	0,201	8,824	1,338	28,943	1,346	0,114
36,543	1,500	0,290	0,140	7,239	1,060	29,773	1,904	0,076
39,531	1,335	0,448	0,283	8,241	1,530	33,627	2,084	0,137
40,312	2,241	0,412	0,245	10,764	1,632	35,306	1,642	0,140
40,838	1,930	0,394	0,167	10,572	1,585	12,515	1,945	0,102
40,535	1,369	0,459	0,291	8,450	1,569	23,354	2,137	0,141
40,309	2,151	0,439	0,186	11,782	1,767	36,049	2,167	0,114
32,844	1,826	0,336	0,200	8,770	1,330	28,766	1,338	0,114
33,273	1,573	0,321	0,136	8,613	1,291	37,394	1,584	0,083
42,109	2,148	0,439	0,186	11,765	1,764	28,821	2,164	0,114
45,450	1,535	0,515	0,326	9,475	1,759	38,662	2,396	0,158
30,207	1,041	0,369	0,093	7,186	1,359	29,249	1,612	0,092
32,566	1,509	0,339	0,117	8,875	1,395	27,520	1,215	0,118
28,766	1,333	0,299	0,103	7,839	1,232	24,309	1,074	0,104
30,974	1,068	0,379	0,096	7,368	1,394	29,992	1,653	0,094
32,061	1,485	0,333	0,115	8,737	1,373	27,093	1,197	0,116
26,533	1,229	0,276	0,095	7,231	1,136	22,422	0,990	0,096
23,438	1,086	0,244	0,084	6,387	1,004	19,806	0,875	0,085
32,013	1,483	0,333	0,115	8,724	1,371	27,053	1,195	0,116
28,664	0,988	0,351	0,089	6,819	1,290	27,755	1,529	0,087
33,185	1,121	0,376	0,238	6,918	1,284	19,388	1,750	0,115
32,877	1,523	0,342	0,118	8,960	1,408	27,783	1,227	0,119
23,035	1,376	0,461	0,292	8,488	1,576	25,795	2,147	0,141
27,209	1,260	0,283	0,098	7,415	1,165	22,994	1,015	0,098
24,213	1,076	0,574	0,155	16,447	2,174	23,372	2,067	0,152
30,786	1,061	0,377	0,095	7,323	1,385	29,810	1,643	0,094
33,191	1,538	0,345	0,119	9,045	1,422	28,048	1,239	0,120
29,318	1,358	0,305	0,105	7,990	1,256	24,776	1,094	0,106
31,568	1,088	0,386	0,098	7,509	1,420	30,568	1,684	0,096
32,676	1,514	0,340	0,117	8,905	1,400	27,613	1,220	0,118
27,042	1,253	0,281	0,097	7,369	1,158	22,852	1,009	0,098
23,887	1,107	0,248	0,086	6,510	1,023	20,186	0,892	0,086
32,627	1,511	0,339	0,117	8,891	1,397	27,572	1,218	0,118
35,396	1,220	0,433	0,109	8,420	1,592	34,274	1,888	0,108
40,978	1,384	0,464	0,294	8,543	1,586	23,941	2,160	0,142
40,599	1,881	0,422	0,146	11,064	1,739	34,309	1,515	0,147
28,444	1,699	0,570	0,360	10,482	1,946	31,853	2,651	0,175
33,600	1,556	0,349	0,121	9,156	1,439	28,394	1,254	0,121
29,899	1,329	0,709	0,191	20,310	2,685	28,860	2,552	0,188
38,017	1,310	0,465	0,118	9,043	1,710	36,811	2,028	0,116
40,986	1,899	0,426	0,147	11,169	1,755	34,635	1,530	0,148
36,204	1,677	0,376	0,130	9,866	1,551	30,594	1,351	0,131
38,982	1,344	0,477	0,121	9,273	1,754	37,746	2,080	0,119
40,350	1,869	0,420	0,145	10,996	1,728	34,098	1,506	0,146
33,393	1,547	0,347	0,120	9,100	1,430	28,219	1,246	0,121
29,497	1,366	0,307	0,106	8,038	1,263	24,927	1,101	0,106
40,289	1,866	0,419	0,145	10,979	1,726	34,047	1,504	0,145
43,709	1,507	0,535	0,135	10,397	1,966	42,323	2,332	0,133
29,044	0,953	0,414	0,109	9,381	1,738	21,644	0,102	0,818

32,436	1,020	0,319	0,109	6,874	1,080	21,315	0,941	0,091
29,781	0,977	0,425	0,111	9,620	1,782	22,584	0,105	0,839
28,652	0,901	0,282	0,096	6,072	0,954	18,828	0,832	0,080
26,428	0,831	0,260	0,089	5,600	0,880	17,367	0,767	0,074
30,250	0,951	0,298	0,102	6,410	1,008	19,878	0,878	0,085
32,476	1,299	0,407	0,139	8,756	1,376	24,151	1,199	0,116
27,560	0,904	0,393	0,103	8,902	1,649	2,081	0,097	0,776
31,914	1,100	0,390	0,099	7,592	1,436	22,061	1,703	0,097
29,382	0,924	0,289	0,099	6,226	0,979	19,308	0,853	0,082
21,475	1,350	0,479	0,121	9,315	1,762	20,234	2,089	0,119
27,101	0,852	0,267	0,091	5,743	0,903	17,809	0,787	0,076
25,314	1,815	0,527	0,918	9,297	1,620	20,464	3,126	0,167
29,601	0,971	0,422	0,111	9,561	1,771	22,059	0,104	0,834
33,058	1,040	0,326	0,111	7,006	1,101	21,724	0,959	0,093
30,353	0,996	0,433	0,113	9,804	1,816	23,017	0,107	0,855
29,201	0,918	0,288	0,098	6,188	0,973	19,189	0,847	0,082
26,935	0,847	0,265	0,090	5,708	0,897	17,700	0,782	0,076
30,830	0,970	0,304	0,104	6,533	1,027	20,260	0,895	0,087
33,099	1,324	0,415	0,141	8,924	1,403	24,615	1,222	0,118
34,033	1,117	0,485	0,127	10,993	2,037	2,570	0,120	0,959
39,409	1,358	0,482	0,122	9,374	1,773	27,242	2,103	0,120
36,282	1,141	0,357	0,122	7,689	1,208	23,842	1,053	0,102
26,519	1,667	0,591	0,150	11,502	2,175	24,986	2,580	0,147
33,466	1,052	0,330	0,112	7,092	1,115	21,992	0,971	0,094
31,260	2,242	0,651	1,134	11,480	2,000	25,270	3,860	0,206
36,553	1,199	0,521	0,137	11,807	2,187	27,240	0,129	1,030
40,822	1,284	0,402	0,137	8,651	1,360	26,826	1,185	0,115
37,481	1,230	0,534	0,140	12,107	2,243	28,423	0,132	1,056
36,059	1,134	0,355	0,121	7,641	1,201	23,696	1,047	0,101
33,260	1,046	0,328	0,112	7,048	1,108	21,857	0,965	0,093
38,070	1,197	0,375	0,128	8,068	1,268	25,018	1,105	0,107
40,872	1,635	0,512	0,175	11,019	1,732	30,395	1,509	0,146
42,026	1,379	0,599	0,157	13,575	2,515	3,174	0,148	1,184
25,517	0,891	0,440	0,123	11,970	2,037	26,251	1,477	0,101
34,127	0,859	0,306	0,152	8,599	1,390	21,925	1,202	0,074
26,165	0,914	0,451	0,127	12,274	2,088	27,142	1,515	0,103
30,145	0,759	0,270	0,134	7,596	1,228	19,367	1,062	0,065
27,805	0,700	0,249	0,124	7,006	1,133	17,864	0,979	0,060
31,826	0,801	0,285	0,141	8,019	1,296	20,447	1,121	0,069
32,861	1,094	0,390	0,193	10,953	1,771	21,928	1,531	0,094
24,213	0,845	0,418	0,117	11,359	1,933	33,300	1,402	0,096
30,685	1,007	0,438	0,115	9,912	1,836	20,000	0,108	0,864
30,913	0,778	0,277	0,137	7,789	1,259	19,860	1,089	0,067
28,808	1,235	0,537	0,141	12,161	2,253	2,843	0,132	1,060
28,514	0,718	0,256	0,127	7,185	1,161	18,319	1,004	0,061
26,006	0,908	0,449	0,126	12,200	2,076	26,755	1,506	0,103
34,782	0,876	0,312	0,155	8,764	1,417	22,346	1,225	0,075
26,667	0,931	0,460	0,129	12,509	2,129	27,663	1,544	0,105
30,723	0,774	0,275	0,137	7,741	1,251	19,738	1,082	0,066
28,339	0,713	0,254	0,126	7,140	1,154	18,206	0,998	0,061

32,437	0,817	0,291	0,144	8,173	1,321	20,839	1,142	0,070
33,492	1,115	0,397	0,197	11,163	1,805	22,349	1,560	0,096
29,900	1,044	0,516	0,145	14,026	2,387	41,120	1,731	0,118
37,891	1,243	0,540	0,142	12,239	2,268	24,697	0,133	1,067
38,173	0,961	0,342	0,170	9,618	1,555	24,525	1,344	0,082
35,574	1,525	0,663	0,174	15,017	2,782	3,511	0,164	1,310
35,210	0,886	0,316	0,156	8,872	1,434	22,621	1,240	0,076
32,114	1,121	0,554	0,155	15,065	2,563	33,038	1,859	0,127
42,950	1,081	0,385	0,191	10,822	1,749	27,594	1,513	0,093
32,929	1,150	0,568	0,159	15,447	2,628	34,160	1,906	0,130
37,939	0,955	0,340	0,169	9,559	1,545	24,374	1,336	0,082
34,994	0,881	0,314	0,155	8,817	1,425	22,482	1,232	0,075
40,055	1,008	0,359	0,178	10,093	1,632	25,733	1,411	0,086
41,357	1,377	0,490	0,243	13,785	2,228	27,597	1,927	0,118
36,922	1,289	0,637	0,179	17,320	2,947	50,778	2,138	0,146
25,499	0,830	0,443	0,119	12,688	1,677	24,955	1,594	0,117
27,873	0,692	0,321	0,109	8,500	1,336	26,357	1,164	0,113
26,146	0,851	0,454	0,122	13,010	1,720	25,813	1,635	0,120
24,621	0,611	0,284	0,097	7,508	1,180	23,282	1,028	0,099
22,710	0,564	0,262	0,089	6,925	1,088	21,475	0,948	0,092
25,994	0,646	0,299	0,102	7,927	1,246	24,580	1,086	0,105
21,478	0,699	0,373	0,101	10,687	1,413	28,467	1,343	0,099
26,664	0,882	0,409	0,139	10,827	1,702	24,732	1,483	0,143
24,196	0,788	0,420	0,113	12,040	1,592	32,070	1,513	0,111
26,958	0,941	0,465	0,130	12,646	2,152	28,234	1,561	0,106
25,248	0,627	0,291	0,099	7,699	1,210	23,875	1,054	0,102
24,236	1,155	0,570	0,160	15,517	2,640	24,268	1,915	0,131
23,289	0,578	0,268	0,091	7,102	1,116	22,022	0,973	0,094
25,988	0,846	0,451	0,122	12,931	1,709	25,433	1,625	0,120
28,408	0,705	0,327	0,111	8,663	1,361	26,863	1,186	0,115
26,648	0,867	0,463	0,125	13,260	1,753	26,308	1,666	0,123
25,093	0,623	0,289	0,098	7,652	1,203	23,728	1,048	0,101
23,146	0,575	0,267	0,091	7,058	1,109	21,887	0,967	0,093
26,493	0,658	0,305	0,104	8,079	1,270	25,052	1,106	0,107
21,890	0,713	0,380	0,103	10,892	1,440	29,013	1,369	0,101
27,175	0,899	0,417	0,142	11,034	1,734	25,207	1,511	0,146
29,879	0,973	0,519	0,140	14,867	1,965	39,601	1,868	0,138
33,289	1,162	0,574	0,161	15,616	2,657	34,865	1,927	0,131
31,178	0,774	0,359	0,122	9,507	1,494	29,482	1,302	0,126
29,928	1,426	0,704	0,198	19,161	3,260	29,968	2,365	0,161
28,758	0,714	0,331	0,113	8,769	1,378	27,194	1,201	0,116
32,091	1,045	0,557	0,150	15,968	2,111	31,406	2,006	0,148
35,080	0,871	0,404	0,138	10,697	1,681	33,172	1,465	0,142
32,906	1,071	0,571	0,154	16,374	2,164	32,487	2,057	0,151
30,987	0,769	0,357	0,122	9,449	1,485	29,301	1,294	0,125
28,581	0,710	0,329	0,112	8,716	1,370	27,027	1,194	0,115
32,715	0,812	0,377	0,128	9,976	1,568	30,935	1,366	0,132
27,031	0,880	0,469	0,127	13,450	1,778	35,827	1,690	0,124
33,557	1,110	0,515	0,175	13,626	2,142	31,127	1,866	0,180
36,896	1,201	0,640	0,173	18,359	2,427	48,902	2,307	0,170

c18:3 n3	CLA9	CLA10	c20:3 n3	c20:4 n6	c20:4 n3	c20:5n3	c22:5	c22:6
0,865	0,405	0,057	0,035	0,364	0,302	0,430	0,615	0,124
0,704	0,270	0,051	0,037	0,144	0,139	0,332	0,211	0,049
0,601	0,231	0,043	0,031	0,123	0,118	0,284	0,180	0,042
0,887	0,416	0,059	0,035	0,374	0,309	0,441	0,630	0,127
0,670	0,257	0,048	0,035	0,137	0,132	0,316	0,201	0,046
0,573	0,220	0,041	0,030	0,118	0,113	0,271	0,172	0,040
0,490	0,188	0,035	0,025	0,100	0,097	0,231	0,147	0,034
0,598	0,230	0,043	0,031	0,123	0,118	0,282	0,179	0,041
0,669	0,257	0,048	0,035	0,137	0,132	0,316	0,201	0,046
0,820	0,385	0,054	0,033	0,346	0,286	0,408	0,583	0,117
0,817	0,313	0,059	0,042	0,167	0,161	0,386	0,245	0,057
1,010	0,667	0,073	0,054	0,159	0,120	0,269	0,241	0,036
0,588	0,226	0,042	0,031	0,121	0,116	0,277	0,176	0,041
0,881	0,413	0,058	0,035	0,371	0,308	0,439	0,626	0,126
0,717	0,275	0,052	0,037	0,147	0,141	0,338	0,215	0,050
0,612	0,235	0,044	0,032	0,126	0,121	0,289	0,184	0,042
0,904	0,424	0,060	0,036	0,381	0,315	0,450	0,642	0,129
0,683	0,262	0,049	0,035	0,140	0,135	0,322	0,205	0,047
0,584	0,224	0,042	0,030	0,120	0,115	0,276	0,175	0,040
0,499	0,192	0,036	0,026	0,102	0,098	0,236	0,150	0,035
0,610	0,234	0,044	0,032	0,125	0,120	0,288	0,183	0,042
0,682	0,262	0,049	0,035	0,140	0,134	0,322	0,204	0,047
0,836	0,392	0,055	0,033	0,352	0,292	0,416	0,594	0,120
0,000	0,000	0,000	0,000	0,000	0,000	0,000	0,000	0,000
1,009	0,387	0,073	0,052	0,207	0,199	0,476	0,303	0,070
1,248	0,824	0,091	0,067	0,196	0,149	0,333	0,298	0,045
0,726	0,279	0,052	0,038	0,149	0,143	0,343	0,218	0,050
1,088	0,510	0,072	0,043	0,459	0,380	0,542	0,774	0,156
0,886	0,340	0,064	0,046	0,182	0,175	0,418	0,266	0,061
0,756	0,290	0,054	0,039	0,155	0,149	0,357	0,227	0,052
1,116	0,523	0,074	0,045	0,470	0,389	0,555	0,793	0,160
0,843	0,323	0,061	0,044	0,173	0,166	0,398	0,253	0,058
0,721	0,277	0,052	0,037	0,148	0,142	0,341	0,216	0,050
0,616	0,236	0,044	0,032	0,126	0,121	0,291	0,185	0,043
0,753	0,289	0,054	0,039	0,154	0,148	0,355	0,226	0,052
0,842	0,323	0,061	0,044	0,173	0,166	0,397	0,252	0,058
1,033	0,484	0,068	0,041	0,435	0,360	0,514	0,734	0,148
0,841	0,406	0,065	0,037	0,359	0,299	0,692	0,561	0,095
0,744	0,340	0,067	0,028	0,166	0,167	0,338	0,224	0,163
0,801	0,335	0,059	0,021	0,175	0,200	0,404	0,286	0,049
0,863	0,416	0,066	0,038	0,368	0,307	0,709	0,576	0,097
0,893	0,373	0,066	0,024	0,195	0,223	0,450	0,318	0,055
0,606	0,277	0,054	0,023	0,135	0,136	0,276	0,183	0,133
0,653	0,273	0,048	0,017	0,143	0,163	0,329	0,233	0,040
0,891	0,373	0,066	0,024	0,195	0,223	0,449	0,318	0,055
0,798	0,385	0,061	0,035	0,341	0,284	0,656	0,533	0,090
0,913	0,428	0,061	0,036	0,385	0,319	0,455	0,649	0,131
0,915	0,383	0,068	0,024	0,200	0,229	0,461	0,327	0,056
1,121	0,525	0,074	0,045	0,472	0,391	0,558	0,797	0,160

0,622	0,284	0,056	0,023	0,138	0,140	0,283	0,187	0,136
0,502	0,193	0,036	0,026	0,103	0,099	0,237	0,151	0,035
0,858	0,413	0,066	0,038	0,366	0,305	0,705	0,572	0,096
0,758	0,347	0,068	0,028	0,169	0,170	0,345	0,228	0,166
0,816	0,341	0,060	0,022	0,179	0,204	0,411	0,291	0,050
0,879	0,424	0,067	0,039	0,375	0,313	0,723	0,587	0,099
0,910	0,380	0,067	0,024	0,199	0,228	0,458	0,325	0,056
0,618	0,282	0,055	0,023	0,138	0,139	0,281	0,186	0,135
0,665	0,278	0,049	0,018	0,146	0,166	0,335	0,237	0,041
0,908	0,380	0,067	0,024	0,199	0,227	0,458	0,324	0,056
0,814	0,392	0,062	0,036	0,347	0,289	0,669	0,543	0,092
1,128	0,529	0,075	0,045	0,475	0,394	0,561	0,802	0,161
1,130	0,473	0,084	0,030	0,247	0,283	0,570	0,403	0,069
1,384	0,649	0,092	0,055	0,583	0,483	0,689	0,984	0,198
0,768	0,351	0,069	0,029	0,171	0,173	0,349	0,231	0,168
0,620	0,238	0,045	0,032	0,127	0,122	0,293	0,186	0,043
1,059	0,510	0,081	0,047	0,452	0,377	0,871	0,707	0,119
0,937	0,428	0,084	0,035	0,208	0,210	0,426	0,282	0,205
1,008	0,422	0,075	0,027	0,221	0,252	0,508	0,360	0,062
1,086	0,523	0,083	0,048	0,463	0,386	0,893	0,725	0,122
1,124	0,470	0,083	0,030	0,246	0,281	0,566	0,401	0,069
0,763	0,349	0,068	0,028	0,170	0,171	0,347	0,230	0,167
0,821	0,343	0,061	0,022	0,180	0,205	0,414	0,293	0,050
1,122	0,469	0,083	0,030	0,246	0,281	0,565	0,400	0,069
1,005	0,484	0,077	0,044	0,429	0,357	0,826	0,671	0,113
0,832	0,497	0,067	0,043	0,311	0,263	0,263	0,458	0,282
0,749	0,406	0,072	0,034	0,156	0,159	0,293	0,210	0,139
0,811	0,374	0,059	0,029	0,157	0,182	0,227	0,217	0,039
0,854	0,509	0,069	0,045	0,319	0,269	0,269	0,469	0,290
0,904	0,417	0,066	0,032	0,175	0,203	0,253	0,242	0,044
0,610	0,331	0,058	0,027	0,127	0,129	0,238	0,171	0,113
0,661	0,305	0,048	0,024	0,128	0,148	0,185	0,177	0,032
0,903	0,416	0,066	0,032	0,174	0,202	0,252	0,241	0,043
0,790	0,471	0,063	0,041	0,295	0,249	0,249	0,434	0,268
0,889	0,429	0,068	0,039	0,379	0,316	0,731	0,593	0,100
0,927	0,428	0,068	0,033	0,179	0,208	0,259	0,248	0,045
1,091	0,526	0,084	0,048	0,466	0,388	0,897	0,728	0,123
0,626	0,339	0,060	0,028	0,131	0,133	0,245	0,176	0,116
0,669	0,280	0,049	0,018	0,146	0,167	0,337	0,239	0,041
0,848	0,506	0,068	0,044	0,317	0,268	0,268	0,466	0,288
0,763	0,414	0,073	0,034	0,159	0,162	0,298	0,215	0,141
0,827	0,381	0,060	0,030	0,160	0,185	0,231	0,221	0,040
0,870	0,519	0,070	0,045	0,325	0,274	0,274	0,478	0,295
0,921	0,425	0,067	0,033	0,178	0,206	0,257	0,246	0,044
0,622	0,337	0,059	0,028	0,130	0,132	0,243	0,175	0,115
0,674	0,311	0,049	0,024	0,130	0,151	0,188	0,180	0,032
0,920	0,424	0,067	0,033	0,178	0,206	0,257	0,246	0,044
0,975	0,582	0,078	0,051	0,365	0,308	0,308	0,536	0,331
1,098	0,529	0,084	0,048	0,469	0,390	0,903	0,732	0,124
1,145	0,528	0,083	0,041	0,221	0,256	0,320	0,306	0,055

1,347	0,649	0,103	0,059	0,575	0,479	1,107	0,899	0,152
0,772	0,419	0,074	0,035	0,161	0,164	0,302	0,217	0,143
0,826	0,346	0,061	0,022	0,181	0,207	0,416	0,295	0,051
1,048	0,625	0,084	0,055	0,392	0,331	0,331	0,576	0,355
0,942	0,511	0,090	0,042	0,197	0,200	0,368	0,265	0,175
1,021	0,471	0,074	0,037	0,197	0,229	0,285	0,273	0,049
1,074	0,641	0,086	0,056	0,402	0,339	0,339	0,591	0,364
1,138	0,525	0,083	0,041	0,220	0,255	0,318	0,304	0,055
0,768	0,416	0,073	0,035	0,160	0,163	0,300	0,216	0,142
0,832	0,384	0,061	0,030	0,161	0,186	0,232	0,222	0,040
1,136	0,524	0,083	0,041	0,219	0,255	0,317	0,304	0,055
1,205	0,719	0,097	0,063	0,450	0,380	0,380	0,662	0,409
0,819	0,565	0,063	0,045	0,257	0,220	0,452	0,382	0,064
0,735	0,440	0,070	0,036	0,147	0,147	0,359	0,201	0,014
0,649	0,389	0,062	0,032	0,130	0,129	0,317	0,177	0,012
0,840	0,579	0,065	0,046	0,264	0,226	0,464	0,391	0,065
0,724	0,434	0,069	0,035	0,145	0,144	0,353	0,198	0,013
0,599	0,359	0,057	0,029	0,120	0,119	0,292	0,164	0,011
0,529	0,317	0,050	0,026	0,106	0,105	0,258	0,145	0,010
0,723	0,433	0,069	0,035	0,145	0,144	0,353	0,197	0,013
0,777	0,536	0,060	0,043	0,244	0,209	0,429	0,362	0,060
0,880	0,525	0,071	0,046	0,329	0,277	0,277	0,484	0,298
0,742	0,445	0,071	0,036	0,149	0,148	0,362	0,203	0,014
1,079	0,644	0,087	0,056	0,403	0,340	0,340	0,593	0,366
0,614	0,368	0,058	0,030	0,123	0,122	0,300	0,168	0,011
1,240	0,819	0,090	0,067	0,195	0,148	0,331	0,296	0,044
0,834	0,576	0,064	0,046	0,262	0,224	0,461	0,389	0,065
0,749	0,449	0,071	0,037	0,150	0,149	0,366	0,205	0,014
0,662	0,397	0,063	0,032	0,133	0,132	0,323	0,181	0,012
0,856	0,590	0,066	0,047	0,269	0,230	0,473	0,399	0,067
0,738	0,442	0,070	0,036	0,148	0,147	0,360	0,202	0,014
0,610	0,366	0,058	0,030	0,122	0,122	0,298	0,167	0,011
0,539	0,323	0,051	0,026	0,108	0,108	0,263	0,147	0,010
0,737	0,441	0,070	0,036	0,148	0,147	0,360	0,201	0,014
0,959	0,662	0,074	0,053	0,301	0,258	0,530	0,447	0,075
1,086	0,648	0,087	0,057	0,406	0,343	0,343	0,597	0,368
0,916	0,549	0,087	0,045	0,184	0,183	0,447	0,250	0,017
1,333	0,795	0,107	0,070	0,498	0,420	0,420	0,733	0,452
0,758	0,454	0,072	0,037	0,152	0,151	0,370	0,207	0,014
1,531	1,011	0,111	0,083	0,241	0,182	0,408	0,365	0,055
1,030	0,711	0,079	0,057	0,324	0,277	0,569	0,480	0,080
0,925	0,554	0,088	0,045	0,185	0,184	0,452	0,253	0,017
0,817	0,490	0,078	0,040	0,164	0,163	0,399	0,223	0,015
1,057	0,729	0,081	0,058	0,332	0,284	0,584	0,493	0,082
0,911	0,546	0,087	0,044	0,183	0,182	0,445	0,249	0,017
0,754	0,452	0,072	0,037	0,151	0,150	0,368	0,206	0,014
0,666	0,399	0,063	0,033	0,133	0,133	0,325	0,182	0,012
0,909	0,545	0,087	0,044	0,182	0,181	0,444	0,249	0,017
1,185	0,817	0,091	0,066	0,372	0,318	0,655	0,552	0,092
0,686	0,629	0,054	0,060	0,139	0,139	0,149	0,253	0,042

0,569	0,341	0,054	0,028	0,114	0,114	0,278	0,156	0,011
0,703	0,645	0,056	0,062	0,142	0,142	0,152	0,260	0,043
0,503	0,301	0,048	0,025	0,101	0,100	0,246	0,137	0,009
0,464	0,278	0,044	0,023	0,093	0,092	0,226	0,127	0,009
0,531	0,318	0,051	0,026	0,106	0,106	0,259	0,145	0,010
0,725	0,435	0,069	0,035	0,145	0,145	0,354	0,198	0,013
0,651	0,597	0,052	0,057	0,132	0,132	0,141	0,240	0,040
0,865	0,597	0,066	0,048	0,272	0,232	0,478	0,403	0,067
0,516	0,309	0,049	0,025	0,103	0,103	0,252	0,141	0,010
1,061	0,732	0,082	0,059	0,333	0,285	0,586	0,495	0,083
0,476	0,285	0,045	0,023	0,095	0,095	0,232	0,130	0,009
1,375	0,645	0,091	0,055	0,580	0,480	0,684	0,978	0,197
0,699	0,641	0,055	0,061	0,141	0,141	0,151	0,258	0,042
0,580	0,348	0,055	0,028	0,116	0,116	0,283	0,159	0,011
0,717	0,657	0,057	0,063	0,145	0,145	0,155	0,265	0,044
0,513	0,307	0,049	0,025	0,103	0,102	0,250	0,140	0,010
0,473	0,283	0,045	0,023	0,095	0,094	0,231	0,129	0,009
0,541	0,324	0,052	0,026	0,108	0,108	0,264	0,148	0,010
0,739	0,443	0,070	0,036	0,148	0,147	0,361	0,202	0,014
0,804	0,737	0,064	0,070	0,163	0,163	0,174	0,297	0,049
1,068	0,737	0,082	0,059	0,335	0,287	0,590	0,498	0,083
0,637	0,382	0,061	0,031	0,128	0,127	0,311	0,174	0,012
1,311	0,904	0,101	0,073	0,412	0,352	0,724	0,611	0,102
0,587	0,352	0,056	0,029	0,118	0,117	0,287	0,161	0,011
1,698	0,796	0,113	0,068	0,716	0,593	0,845	1,207	0,243
0,863	0,792	0,068	0,076	0,175	0,175	0,187	0,319	0,052
0,717	0,429	0,068	0,035	0,144	0,143	0,350	0,196	0,013
0,885	0,812	0,070	0,078	0,179	0,179	0,192	0,327	0,054
0,633	0,379	0,060	0,031	0,127	0,126	0,309	0,173	0,012
0,584	0,350	0,056	0,029	0,117	0,116	0,285	0,160	0,011
0,668	0,400	0,064	0,033	0,134	0,133	0,326	0,183	0,012
0,913	0,547	0,087	0,045	0,183	0,182	0,446	0,249	0,017
0,992	0,910	0,079	0,087	0,201	0,201	0,215	0,367	0,060
0,913	0,653	0,067	0,055	0,152	0,123	0,249	0,231	0,037
0,602	0,451	0,046	0,042	0,119	0,099	0,659	0,151	0,014
0,936	0,669	0,068	0,057	0,156	0,126	0,255	0,237	0,038
0,531	0,398	0,041	0,037	0,105	0,087	0,583	0,133	0,013
0,490	0,367	0,038	0,034	0,097	0,081	0,537	0,123	0,012
0,561	0,420	0,043	0,039	0,111	0,092	0,615	0,140	0,013
0,766	0,574	0,059	0,053	0,152	0,126	0,840	0,192	0,018
0,866	0,619	0,063	0,052	0,144	0,117	0,236	0,219	0,035
0,724	0,665	0,057	0,063	0,147	0,147	0,157	0,268	0,044
0,545	0,408	0,042	0,038	0,108	0,090	0,597	0,136	0,013
0,889	0,815	0,070	0,078	0,180	0,180	0,193	0,329	0,054
0,503	0,377	0,039	0,035	0,100	0,083	0,551	0,126	0,012
0,930	0,665	0,068	0,056	0,155	0,125	0,253	0,236	0,037
0,613	0,459	0,047	0,042	0,121	0,101	0,672	0,154	0,015
0,954	0,682	0,070	0,058	0,159	0,128	0,260	0,242	0,038
0,542	0,406	0,042	0,038	0,107	0,089	0,594	0,136	0,013
0,500	0,374	0,039	0,035	0,099	0,082	0,548	0,125	0,012

0,572	0,428	0,044	0,040	0,113	0,094	0,627	0,143	0,014
0,781	0,585	0,060	0,054	0,155	0,128	0,856	0,196	0,019
1,070	0,765	0,078	0,065	0,178	0,144	0,291	0,271	0,043
0,895	0,821	0,071	0,078	0,181	0,181	0,194	0,331	0,054
0,673	0,504	0,052	0,047	0,133	0,111	0,738	0,168	0,016
1,098	1,007	0,087	0,096	0,222	0,222	0,238	0,406	0,067
0,621	0,465	0,048	0,043	0,123	0,102	0,680	0,155	0,015
1,149	0,822	0,084	0,070	0,191	0,155	0,313	0,291	0,046
0,757	0,567	0,058	0,052	0,150	0,124	0,830	0,190	0,018
1,178	0,842	0,086	0,071	0,196	0,158	0,321	0,299	0,047
0,669	0,501	0,052	0,046	0,133	0,110	0,733	0,167	0,016
0,617	0,462	0,048	0,043	0,122	0,101	0,676	0,154	0,015
0,706	0,529	0,054	0,049	0,140	0,116	0,774	0,177	0,017
0,964	0,723	0,074	0,067	0,191	0,158	1,057	0,241	0,023
1,321	0,945	0,096	0,080	0,219	0,178	0,360	0,335	0,053
0,956	0,632	0,070	0,052	0,150	0,114	0,255	0,228	0,034
0,704	0,422	0,067	0,034	0,141	0,140	0,344	0,192	0,013
0,981	0,648	0,071	0,053	0,154	0,117	0,261	0,234	0,035
0,622	0,373	0,059	0,030	0,125	0,124	0,304	0,170	0,012
0,574	0,344	0,055	0,028	0,115	0,114	0,280	0,157	0,011
0,657	0,393	0,063	0,032	0,132	0,131	0,321	0,179	0,012
0,806	0,532	0,059	0,043	0,127	0,096	0,215	0,192	0,029
0,897	0,537	0,085	0,044	0,180	0,179	0,438	0,245	0,017
0,908	0,599	0,066	0,049	0,143	0,108	0,242	0,217	0,033
0,964	0,690	0,070	0,058	0,160	0,130	0,263	0,244	0,039
0,638	0,382	0,061	0,031	0,128	0,127	0,311	0,174	0,012
1,183	0,846	0,086	0,072	0,197	0,159	0,322	0,300	0,047
0,588	0,352	0,056	0,029	0,118	0,117	0,287	0,161	0,011
0,975	0,644	0,071	0,053	0,153	0,116	0,260	0,233	0,035
0,718	0,430	0,068	0,035	0,144	0,143	0,350	0,196	0,013
1,000	0,660	0,073	0,054	0,157	0,119	0,266	0,239	0,036
0,634	0,380	0,060	0,031	0,127	0,126	0,309	0,173	0,012
0,585	0,350	0,056	0,029	0,117	0,117	0,285	0,160	0,011
0,669	0,401	0,064	0,033	0,134	0,133	0,327	0,183	0,012
0,821	0,542	0,060	0,044	0,129	0,098	0,219	0,196	0,029
0,914	0,548	0,087	0,045	0,183	0,182	0,446	0,250	0,017
1,121	0,740	0,081	0,060	0,176	0,134	0,299	0,267	0,040
1,191	0,852	0,087	0,072	0,198	0,160	0,324	0,302	0,048
0,788	0,472	0,075	0,038	0,158	0,157	0,385	0,215	0,015
1,461	1,045	0,107	0,088	0,243	0,197	0,398	0,370	0,059
0,726	0,435	0,069	0,035	0,146	0,145	0,355	0,199	0,014
1,204	0,795	0,087	0,065	0,189	0,143	0,321	0,287	0,043
0,886	0,531	0,084	0,043	0,178	0,177	0,433	0,242	0,016
1,234	0,815	0,090	0,067	0,194	0,147	0,329	0,295	0,044
0,783	0,469	0,075	0,038	0,157	0,156	0,382	0,214	0,015
0,722	0,433	0,069	0,035	0,145	0,144	0,352	0,197	0,013
0,826	0,495	0,079	0,040	0,166	0,165	0,403	0,226	0,015
1,014	0,670	0,074	0,055	0,159	0,121	0,270	0,242	0,036
1,129	0,676	0,107	0,055	0,226	0,225	0,551	0,308	0,021
1,384	0,914	0,101	0,075	0,217	0,165	0,369	0,330	0,050
